# Pentatwinned AuAg Nanorattles with Tailored Plasmonic Properties for Near-Infrared Applications

**DOI:** 10.1021/acs.chemmater.4c01443

**Published:** 2024-09-13

**Authors:** Daniel García-Lojo, Sergio Rodal-Cedeira, Sara Núñez-Sánchez, Daniel Arenas-Esteban, Lakshminarayana Polavarapu, Sara Bals, Jorge Pérez-Juste, Isabel Pastoriza-Santos

**Affiliations:** † CINBIO, Universidade de Vigo, Departamento de Química Física, Campus Universitario As Lagoas, Marcosende, 36310 Vigo, Spain; ‡ Galicia Sur Health Research Institute (IIS Galicia Sur), 36310 Vigo, Spain; § Centro de Física das Universidades do Minho e do Porto (CF-UM-UP), Universidade do Minho, 4710-057 Braga, Portugal; ∥ EMAT, University of Antwerp, Groenenborgerlaa 171, 2020 Antwerp, Belgium

## Abstract

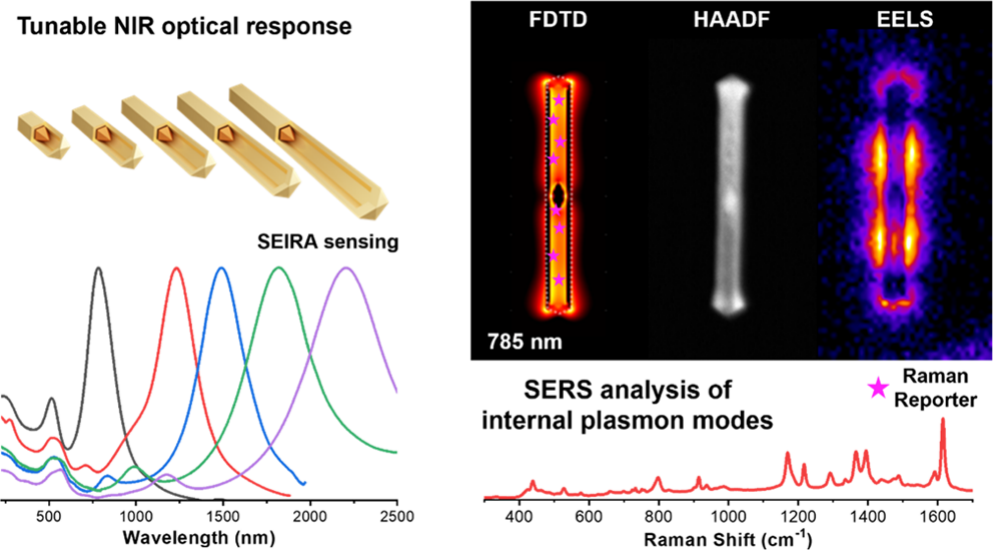

Noble metal nanoparticles, particularly gold and silver nanoparticles, have garnered significant attention due to their ability to manipulate light at the nanoscale through their localized surface plasmon resonance (LSPR). While their LSPRs below 1100 nm were extensively exploited in a wide range of applications, their potential in the near-infrared (NIR) region, crucial for optical communication and sensing, remains relatively underexplored. One primary reason is likely the limited strategies available to obtain highly stable plasmonic nanoparticles with tailored optical properties in the NIR region. Herein, we synthesized AuAg nanorattles (NRTs) with tailored and narrow plasmonic responses ranging from 1000 to 3000 nm. Additionally, we performed comprehensive characterization, employing advanced electron microscopy and various spectroscopic techniques, coupled with finite difference time domain (FDTD) simulations, to elucidate their optical properties. Notably, we unveiled the main external and internal LSPR modes by combining electron energy-loss spectroscopy (EELS) with surface-enhanced Raman scattering (SERS). Furthermore, we demonstrated through surface-enhanced infrared absorption spectroscopy (SEIRA) that the NRTs can significantly enhance the infrared signals of a model molecule. This study not only reports the synthesis of plasmonic NRTs with tunable LSPRs over the entire NIR range but also demonstrates their potential for NIR sensing and optical communication.

## Introduction

In recent decades, noble metal nanoparticles, particularly made of gold and silver, have attracted considerable attention due to their ability to manipulate light at the nanoscale through localized surface plasmon resonance (LSPR). This arises from the collective oscillations of conduction electrons in response to incident light,^1,2^ making plasmonic nanoparticles highly attractive for a myriad of applications across diverse fields including biosensing, imaging, drug delivery, photocatalysis, and optical devices.^3−5^

Most of the explored applications are generally based on plasmonic nanostructures with LSPRs below 1100 nm. In contrast, applications associated with nanoparticles with optical properties above 1100 nm, such as optical communication, fiber optics, data transmission, or telecommunication, have received less attention.^6^ Recently, the integration of near-infrared (NIR) plasmonic structures with optical fibers has opened up possibilities for sensing applications,^6−8^ and their integration with communication optical fibers is of great interest (optimized for operation in the 1500 to 1600 nm region). Another intriguing and relatively underexplored field of application for NIR-responsive plasmonic metal nanoparticles is surface-enhanced infrared absorption (SEIRA) spectroscopy.^9−13^ This is based on the enhanced IR absorption of a molecule by several orders of magnitude upon interaction with the highly intense near electromagnetic fields generated on a plasmonic surface. This enhancement is proportional to the square of the electromagnetic field (*E*^2^) and consequently depends on the optical properties of the plasmonic nanostructures in the NIR region.^14−16^

The disparity in the applicability of metal nanoparticles, as a function of the LSPR, can be attributed to the challenges in synthesizing colloidal plasmonic nanoparticles with tunable and well-defined plasmonic properties in certain spectral regions. For instance, the only morphologies that have shown the capability to extend their plasmonic response into the NIR region,^17^ or even in the mid-IR region are the ones with high anisotropy such as nanorods or bipyramids with high aspect ratios.^12,13^ El-Sayed and co-workers^18^ demonstrated empirically that in the case of gold nanorods, the LSPR wavelength (λ_LSPR_) depends on their aspect ratio (AR), thus λ_LSPR_ = 420 + 95 *×* AR, highlighting the requirement of nanostructures with large ARs to reach the IR region.

Despite different protocols reported for obtaining Au nanorods and bipyramids, it is still challenging to synthesize nanoparticles with narrow and tunable LSPR above 1200 nm.^17^ Vigderman et al.^19^ synthesized gold nanorods with LSPR up to 1250 nm by employing hydroquinone as a reducing agent. Sánchez-Iglesias et al.^20^ obtained highly monodispersed pentatwinned gold nanorods and bipyramids with tunable LSPR up to 1276 nm (AR of 6.9) and 1796 (AR of 5.3) nm, respectively. Rod shaped Au nanocapsules with LSPR from 700 to 1500 nm as a function of the AR (from 2.8 to 6.5) were reported by Singh et al.^8^ High aspect ratio Au nanorods and nanonails (AR of 10 to 60) with LSPR above 1600 nm were prepared by Yin et al.^13^ although the optical response was rather broad. In the case of Ag, Mayer et al.^21^ demonstrated a synthetic protocol to obtain pentatwinned core–shell Au@Ag nanorods with tunable LSPR response up to 2170 nm (AR of 13). However, the inherent tendency of silver to undergo oxidation poses a significant limitation for practical applications, particularly in terms of long-term stability. The oxidation process gradually diminishes the plasmonic properties of silver shells, making them unsuitable for sustained use in the NIR region.

Herein, we employ a wet-chemistry strategy that couples galvanic replacement and chemical reduction for the synthesis of AuAg nanorattles (NRTs), hollow AuAg nanoparticles containing an Au nanorod/bipyramid, with tailored and narrow plasmonic response in the NIR region (from 1000 to 2500 nm). To get insight into their plasmonic properties, the AuAg NRT are fully characterized by combining experimental techniques such as advanced electron microscopy and a range of spectroscopic techniques including UV–Vis-NIR absorption, electron energy-loss spectroscopy (EELS), and energy-dispersive X-ray spectroscopy (EDS) with simulation tools like the finite difference time domain simulations (FDTD). Furthermore, the plasmon modes localized in the nanorattle interior are analyzed by surface-enhanced Raman scattering (SERS) spectroscopy by labeling the void with a Raman reporter. Finally, we demonstrate the ability of these nanostructures to enhance the IR signal of a molecule (SEIRA).

## Results and Discussion

**Figure 1:**
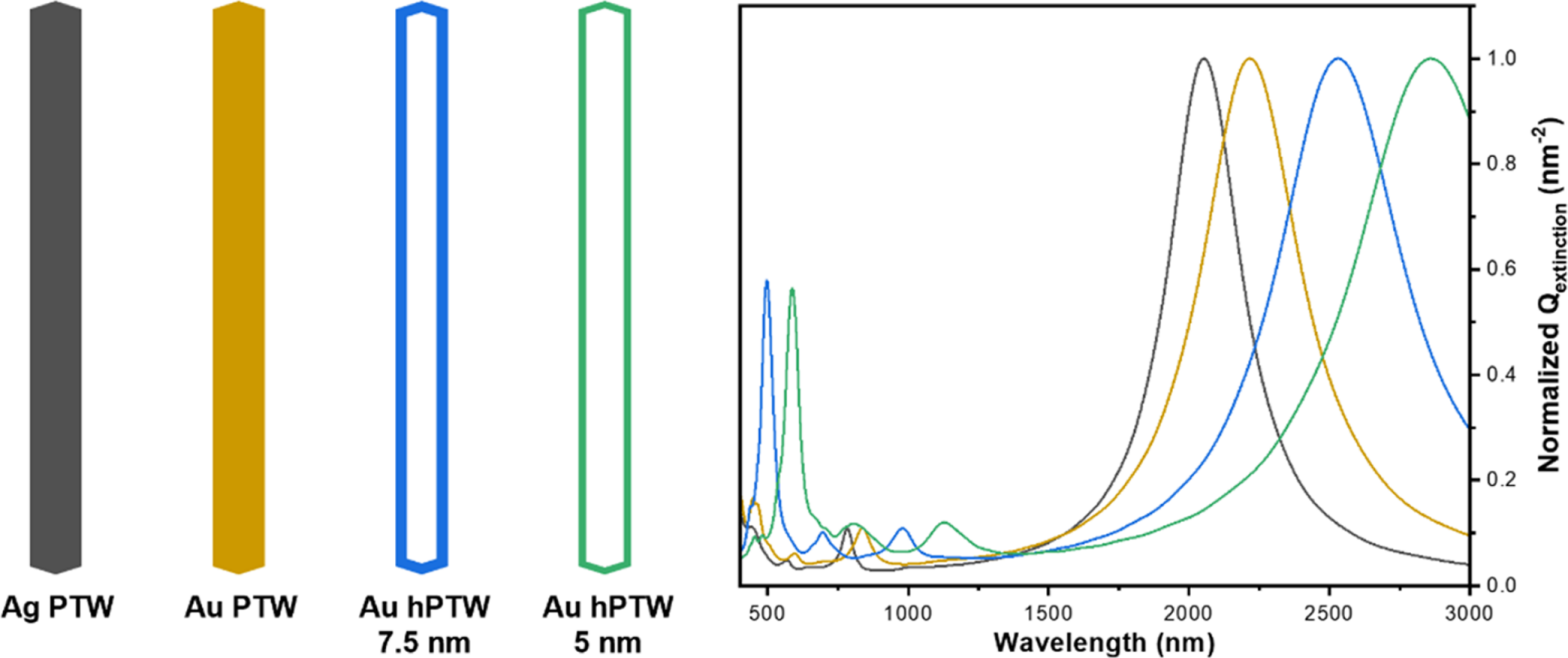
Simulated extinction properties of different gold or silver nanostructures. (left) Schematics of a pentatwinned Ag nanorod (Ag PTW, black), a pentatwinned Au nanorod (Au PTW, yellow), and a hollow Au PTW with a thickness shell of 7.5 nm (blue) and 5 nm (green). All nanostructures have the same overall dimensions (385 nm × 35 nm, aspect ratio of 11). (right) Corresponding simulated extinction cross-section spectra. The spectra were normalized at maximum for comparison.

First, we modeled the extinction spectra of long NRs of Ag and Au along with Au hollow NRs of different wall thicknesses to explore the feasibility of tuning their LSPR in the NIR region. Figure 1 presents a scheme of four different nanostructures: a pentatwinned Ag nanorod (Ag PTW), a pentatwinned Au nanorod (Au PTW), and two hollow pentatwinned Au nanorods (Au hPTW) with different shell thickness (5 and 7.5 nm) and their FDTD simulated extinction cross-section spectra. These nanostructures exhibit the same overall dimensions, 385 nm in length × 35 nm in width, and an aspect ratio of 11. The simulations reveal that the presence of void space in the interior of the Au hPTW induces a red-shift in the dipolar LSPR compared to Au or Ag PTWs. Moreover, the extent of this red-shift depends on the shell thickness (Figure 1 and Figure S1A in the Supporting Information (SI)), being larger for thinner shells or larger void spaces inside the nanostructure.^22^ The simulations also indicate that the inclusion of a gold nanoparticle (core) within the hPTW, hereafter Au nanorattles (Au NRTs), does not alter the energy of the dipolar LSPR mode (Figure S1B), but it generates additional plasmon modes resulting from the interaction between the shell and the core (Figure S1C). These simulations highlight the potential of Au NRT as promising structures for achieving precise and narrow optical properties in the NIR region. Importantly, these properties can be precisely controlled by adjusting the nanostructure’s aspect ratio and shell thickness.

**Figure 2:**
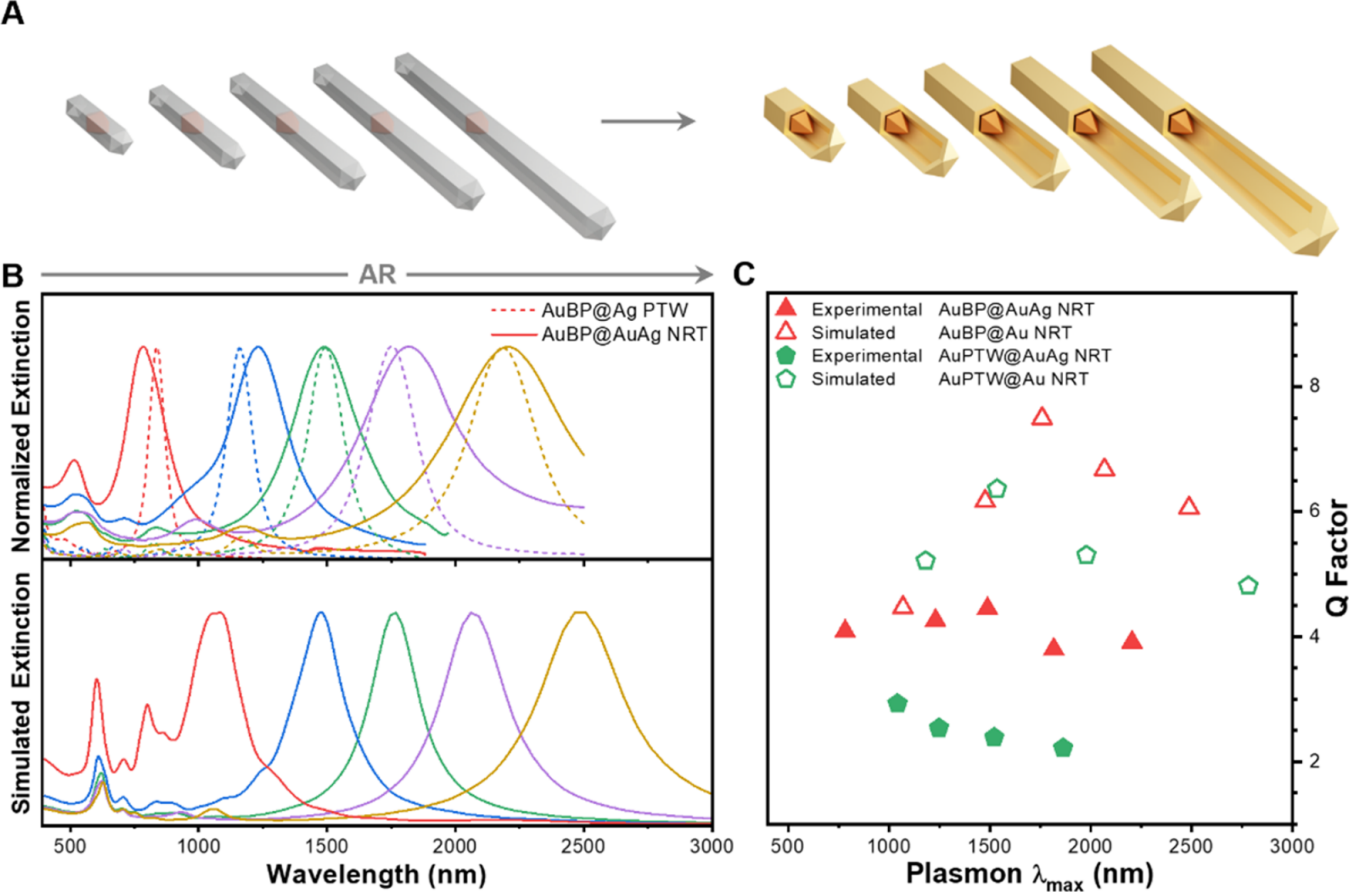
Synthesis of AuAg NRTs with a pentatwinned Au bipyramid (Au BP) core with tailored NIR optical properties. (A) Schematic representation of the synthesis of AuAg NRTs with an AuBP core (AuBP@AuAg NRTs) from Ag PTWs containing an Au BP core (AuBP@Ag PTWs). (B) Top panel: Normalized extinction spectra of AuBP@Ag PTWs with different aspect ratios (dotted line) and the corresponding AuBP@AuAg NRTs (solid line). Bottom panel: corresponding simulated extinction spectra of Au NRTs with an Au BP core (AuBP@Au NRTs). The aspect ratios of the final AuAg NRTs are 3.1 (red), 5.1 (blue), 6.8 (green), 8.6 (violet), and 11.0 (yellow). (C) Comparison of the quality (Q) factor obtained experimentally (solid) and through FDTD calculations (open) for AuAg NRT with an Au BP core (triangle) or Au PTW core (pentagon).

Inspired by the interesting theoretical observations, we developed a synthesis method for NRTs with narrow and tunable optical properties across the entire NIR region. This is achieved through the synthesis of AuAg NRTs of different aspect ratios through the galvanic replacement combined with chemical reduction using Ag PTWs of different aspect ratios as sacrificial templates (Figure 2A). First, cetyltrimethylammonium chloride (CTAC) stabilized Ag PTWs were obtained by preferential growth of silver, as previously reported.^20,23,24^ Two different seeds were used for this purpose, 48.5 nm x 22.5 nm Au bipyramids (Au BPs, Figures S2–S3) and 71.7 nm x 24.5 nm Au PTW (Figures S4–S6). The optical properties of the resulting Ag PTW nanoparticles, obtained from Au BP (AuBP@Ag PTWs, Figure 2B) or Au PTW (AuPTW@Ag PTWs, Figure S7), exhibited the longitudinal LSPR ranging from 750 to 2100 nm as a function of the aspect ratio. Next, both types of NPs were used as sacrificial templates for obtaining AuAg NRTs. In a typical synthesis, 0.5 mM HAuCl_4_ was gradually pumped into a solution containing the Ag PTW, cetyltrimethylammonium bromide (CTAB), and ascorbic acid (see further details in the Experimental section). The process was monitored by UV-Vis-NIR absorption spectroscopy observing the Ag oxidation with the gradual addition of Au salt (see Figure S8 for AuBP@Ag PTW and Figure S9 for AuPTW@Ag PTW in the SI). Interestingly, the optical properties of the resulting nanoparticles closely resembled those exhibited by the corresponding Ag PTW, observing only slight shifts and broadening in the longitudinal LSPRs (Figures 2B and Figure S7 in the SI). Besides, we noticed that the longitudinal LSPR bands for AuPTW@Ag PTW (Figure S7 in the SI) were broader compared to those observed for AuBP@Ag PTW (Figure 2B), resulting in a lower quality (Q) factor (Figure 2C). It should be noted that the aspect ratio of the Au core does not alter the energy of the dipolar LSPR mode (Figure S1B), but it generates additional plasmon modes resulting from the interaction between the shell and the core (Figure S1C).

**Figure 3:**
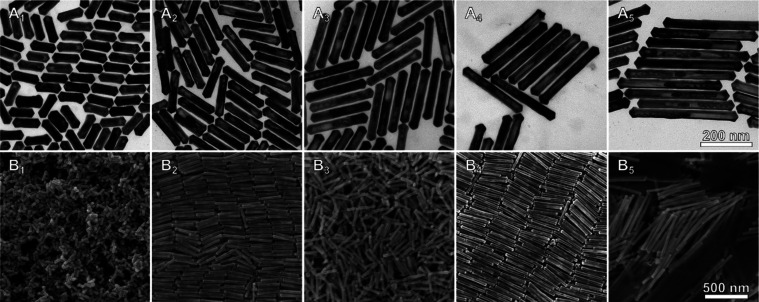
TEM and SEM characterization of AuBP@AuAg NRTs with different aspect ratios. (A) TEM and (B) SEM characterization of AuBP@AuAg NRTs with aspect ratios of: 3.1 (1), 5.1 (2), 6.8 (3), 8.6 (4), and 11.0 (5). In all TEM or SEM images, the scale bar is the same for better comparison.

To understand the relation between morphology/composition and optical features of these nanoparticles, a full electron microscopy characterization was performed. TEM and SEM analysis of the NRTs obtained from AuBP@Ag PTWs (Figure 3 and Figure S10 in the SI) indicated that the resulting nanoparticles resembled the AuBP@Ag PTWs morphology. Besides, the presence of void space between the gold core and the outer shell suggested the formation of NRTs. Furthermore, the TEM analysis (Table S1 in the SI) showed that the aspect ratios of the NRTs were smaller compared to those of corresponding AuBP@Ag PTWs. Finally, the thickness of the NRT shell was estimated considering the average dimensions of the AuBP@Ag PTWs and resulting NRTs, yielding values between 5 and 10 nm under the employed experimental conditions. Table S1 in the SI summarizes the data obtained from the optical and structural analysis of AuBP@Ag PTWs and AuBP@AuAg NRTs. The Au shell thickness could be easily increased by further reduction of Au precursor in the reaction (see Figure S11 and Table S2 in the SI), although this would lead to a further decrease of the aspect ratio and therefore a blue-shift of the main LSPR. Thus, both the aspect ratio of sacrificial AuBP@Ag PTWs and the NRT shell thickness are the factors determining the optical properties of the NRTs. Similar results were also obtained when AuPTW@Ag PTWs were used as sacrificial templates (Figure S12 and Table S3 in the SI).

**Figure 4:**
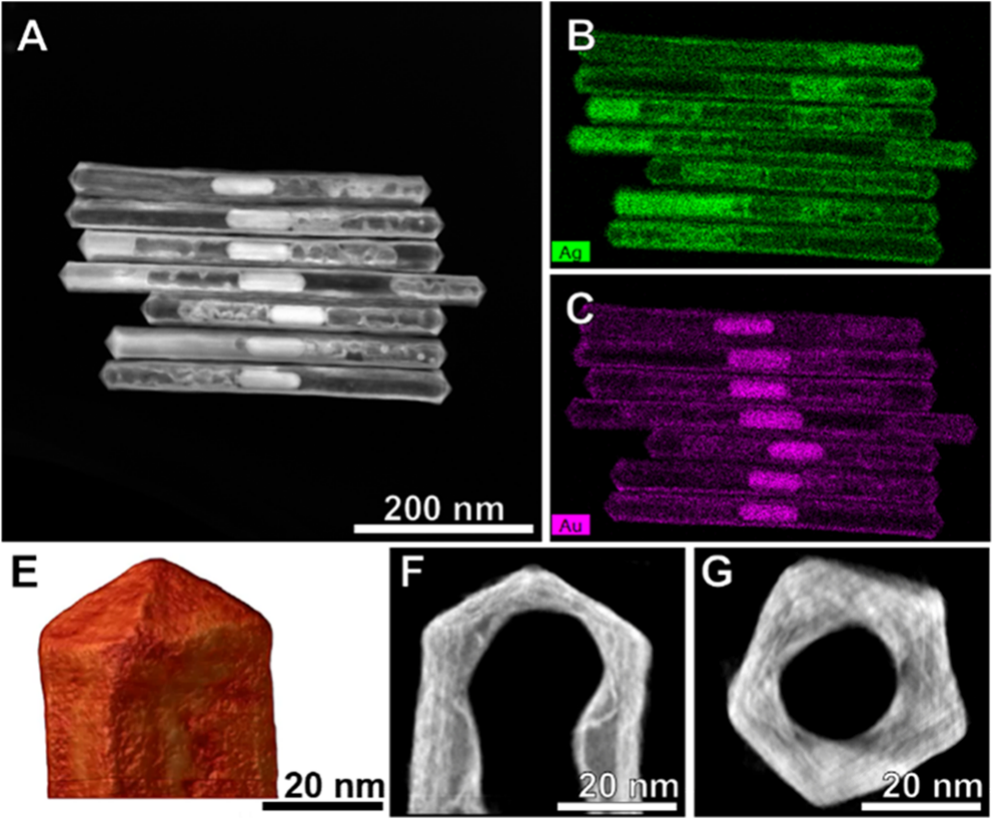
Advanced electron microscopy characterization of AuAg NRTs obtained from AuPTW@Ag PTWs. (A) HAADF-STEM image of AuAg NRTs. (B–C) Corresponding EDS maps showing the spatial distribution of Ag (B) and Au (C). (E) HAADF-STEM tridimensional tomography of a AuAg NRT tip. (F–G) Corresponding orthoslices obtained from the side (F) and the top view (G).

Furthermore, the crystallinity and composition of the NRTs were further investigated by high-angle annular dark-field scanning transmission electron microscopy (HAADF-STEM) and energy-dispersive X-ray spectroscopy (EDS). As shown in Figure 4, the HAADF-STEM images combined with 2D EDS mapping of NRTs obtained from AuPTW@Ag PTWs, indicate that the silver oxidation during the galvanic replacement process was not complete as some silver remained. Thus, the shell of the NRTs is composed of an AuAg alloy with increasing Au content toward the outer surface. Moreover, the 3D tomography reconstruction (Figure 4E–G and Figure S13A–B in the SI) revealed that the NRTs retained the pentatwinned structure of sacrificial templates. Analogous results were obtained for NRTs synthesized from the AuBP@Ag PTWs (Figures S13C–D and S14 in the SI). Interestingly, Ag remained inside the NRTs even after further overgrowth (Figure S15 in the SI). In summary, the characterization of both types of NRTs confirmed the preservation of the pentatwinned structure, as well as the presence of residual silver in the shell. Therefore, from here onward, we have used the nomenclature AuAg NRTs when referring to NRTs, and AuPTW@AuAg NRTs or AuBP@AuAg NRTs when we need to specify the AuPTW or AuBP core, respectively.

The presence of Ag in the AuAg NRTs motivated us to study their chemical stability against oxidation. As revealed in Figure S16 in the SI, despite the presence of Ag in the outer shell of the nanoparticles, AuAg NRTs exhibit remarkable stability in oxidizing environments, such as solutions containing 1% (w/w) hydrogen peroxide, even after 24 h incubation. This contrasts with the behavior of AuBP@Ag PTWs, in which silver oxidation occurs within minutes in the presence of 0.1% (w/w) hydrogen peroxide, leaving only the gold core intact (Figure S16 in the SI).

Upon establishing the structure and dimensions of AuAg NRTs, we conducted a full analysis of their optical response. First, we simulated their extinction spectra via FDTD using as a model an Au NRT with either an Au BP or Au PTW core, with dimensions similar to those obtained experimentally based on TEM analysis (Tables S1 and S3 in the SI). The thickness of the NRT shell were 5 and 7 nm for AuAg NRTs obtained from AuBP@Ag PTWs or AuPTW@ Ag PTWs, respectively. This values represent the average shell thickness, estimated as the difference between the width of the AuAg NRTs and the width of the AuBP@Ag PTWs or AuPTW@Ag PTWs used as templates, across different aspect ratios (Tables S1 and S3 in the SI). It is worth mentioning that we did not consider any Ag content in the NRT during the simulations. The simulations of Au NRTs showed good alignment with the experimental data of both AuBP@AuAg NRTs (Figure 2B) and AuPTW@AuAg NRTs (Figure S7 in the SI**)**. Nevertheless, differences in the longitudinal LSPR position were observed, which could mainly be attributed to variations in shell thickness and composition (no content of Ag was considered in the simulations), given the high sensitivity of the longitudinal LSPR to these parameters.^25^ Importantly, the simulations of the extinction of an Au NRT with an Au PTW core confirmed that the broader optical response is an intrinsic property of the nanostructure morphology and not an experimental artifact. This agreement is further substantiated by the values of the Q factors (see Figure 2C). The difference in the trends observed in the experimental and calculated Q factors may be attributed to the increasing polydispersity of the NRTs as the aspect ratio increases (Tables S1 and S3 in the SI).

Next, we performed a single particle EELS analysis of AuBP@AuAg NRTs to investigate the origin of their LSPR modes and the spatial distribution of their associated near electromagnetic fields. The average EELS spectrum of this AuAg NRT agrees with the extinction spectrum, revealing the presence of four main LSPRs at 0.8, 1.4, 2.0, and 2.4 eV (corresponding to 1500, 886, 620, and 517 nm, respectively) (Figure S17A in SI). It is important to note that the LSPR at 0.8 eV is obscured by the Zero-loss energy contribution, making its EELS characterization very challenging. Additionally, electron scattering from the gold surface limits the experimental characterization of the inner part of the nanostructure. Similar optical features (0.60, 1.33, 1.90, and 2.01 eV corresponding to 2050, 932, 653, and 617 nm, respectively) were obtained by simulating an Au NRT of the same structure and dimensions excited with unpolarized light (Figure S17B in SI).

**Figure 5:**
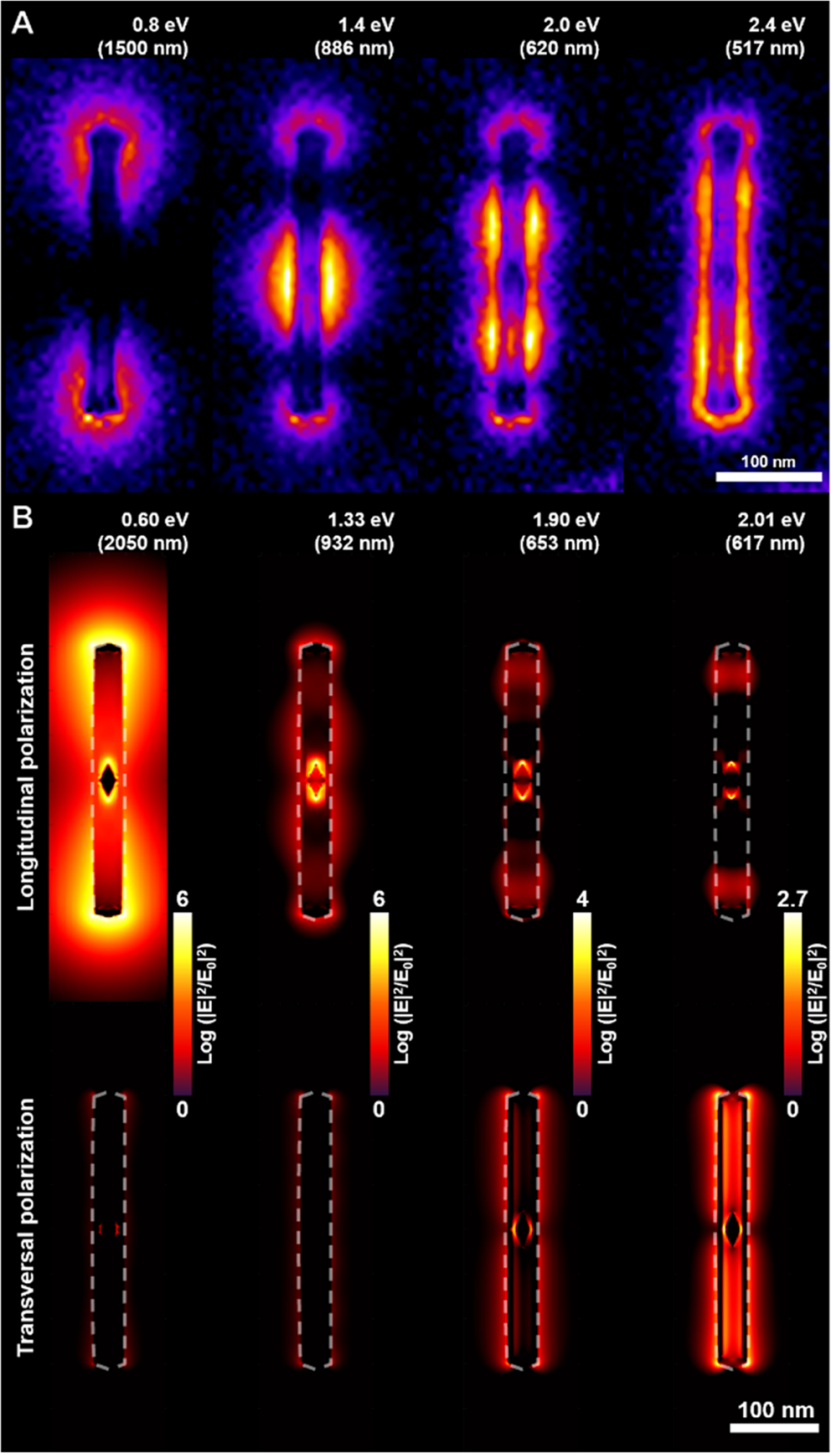
Experimental and FDTD simulated near-field distribution on individual NRTs upon excitation with different energies. (A) Normalized EELS distribution map acquired after excitation of an AuBP@AuAg NRT at 0.8, 1.4, 2.0, and 2.4 eV, as indicated. The color scale is represented in the logarithm scale. (B) FDTD simulated electric field distribution maps of an AuBP@Au NRT at 0.6, 1.33, 1.90, and 2.01 eV for longitudinal (top) and transversal (bottom) excitation. The color scale is represented in a logarithm scale and is the same for each energy. The dashed line represents the outer contour of the nanostructure.

Additionally, the corresponding near-field distribution maps were recorded through EELS to identify these main LSPR modes. The spatial distribution of plasmon modes shown in Figure 5A indicates that the peaks at 0.8 eV (1500 nm), 1.4 eV (886 nm), and 2.0 eV (620 nm) are likely associated with the dipole-, quadrupole- and octupole-like modes of the longitudinal LSPR, respectively. Finally, the near-field resonance obtained at 2.4 eV (517 nm) could be attributed to a mixture of different LSPR modes. But the reality is much more complicated, as the high nanoparticle anisotropy, the internal void space, and the Au core contribute jointly to the optical response rather complex with hybrid modes resulting from the coupling between different metal surfaces inside and outside the shell or polarization dependence of LSPR modes. Nevertheless, through EELS we are not able to visualize the modes localized in the inner part of the NRT due to the thickness of the AuAg shell.

The complex nature of their optical response was evidenced in the near-field distribution maps simulated by FDTD for transversal and longitudinal polarization (Figures 5B, and Video 1) using as a model an Au NRT with similar morphology and dimensions to the one characterized by EELS. As depicted in Figure 5B, the peak at 0.6 eV (2050 nm) is purely due to dipolar longitudinal LSPR, which is mainly localized on the outer surface, but also to some extent on the surface of the Au BP core. Nevertheless, the peak at 1.33 eV (932 nm) can primarily be attributed to longitudinal hybrid modes, among which the mode confined on the Au bipyramid core surface dominates. Then, the range between 1.4 eV (653 nm) and 1.9 eV (617 nm) corresponds to the longitudinal and transversal hybrid modes that localized on the surface of Au BP core at low energies and in the void space at high ones (Video 1). Finally, the peak at 2.01 eV (617 nm) can be ascribed to the transversal LSPR modes that are uniformly localized along the void space and at the hot spot between the Au BP core and the NRT shell. Note that in Figure 5A, the EELS maps are obtained without controlling polarization, while the electric field distributions correspond to longitudinal and transversal polarization. The electric field distribution for unpolarized conditions is provided in Figure S18 in the SI.

In summary, the longitudinal hybrid modes dominated at lower energies and were confined to the NRT and Au BP core tips, while the transversal ones dominated at high energies and were distributed in the inner void space. Since we were not able to resolve the modes confined to the interior of the AuAg NRTs by EELS, we propose to achieve this by SERS spectroscopy. The structure of the AuAg NRTs enables the labeling of their void space with a Raman-active molecule and by recording its SERS features it would be possible to get information about the internal plasmon modes. We anticipate that only the internal SERS field observed in the simulations would impact the Raman signal enhancement.

**Figure 6:**
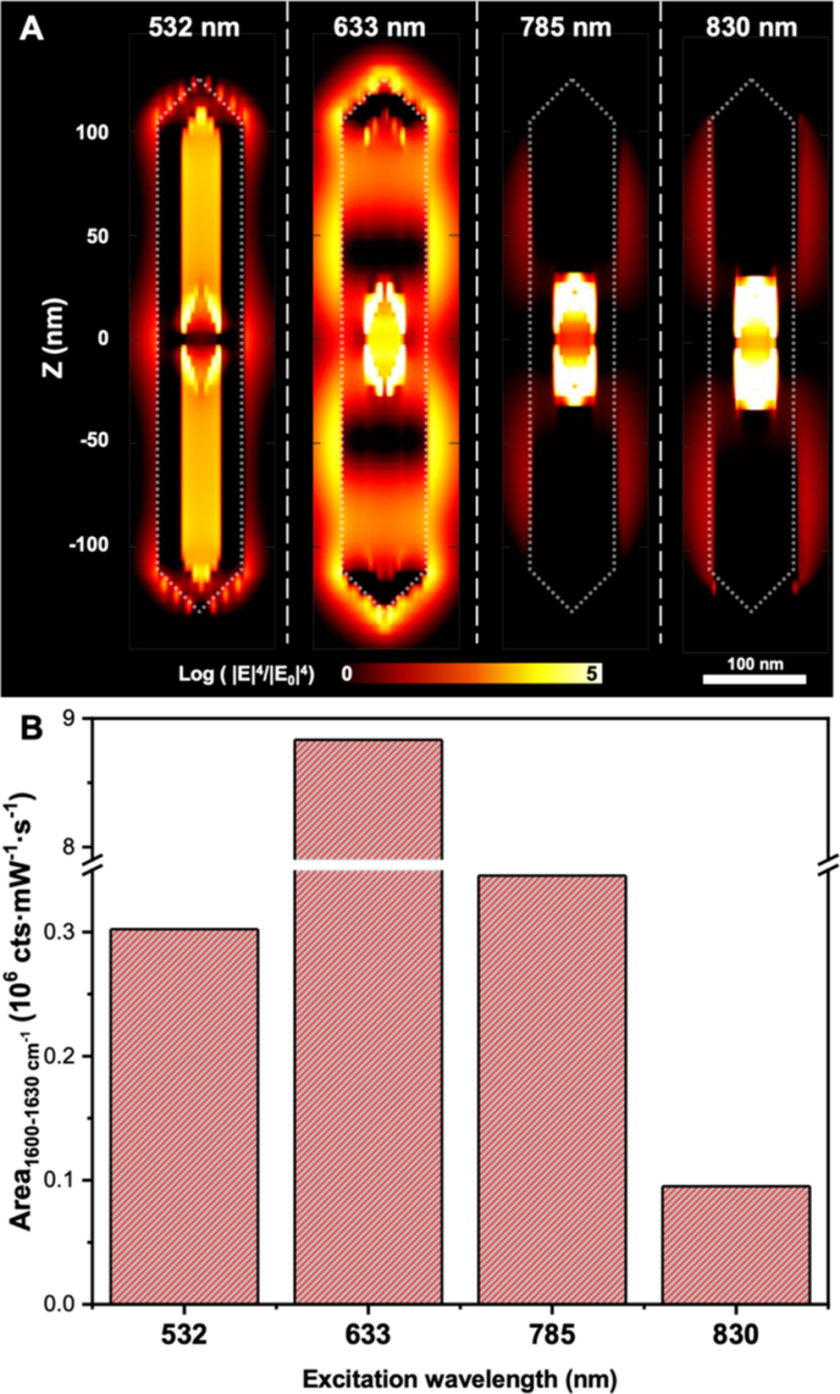
Simulated and experimental SERS analysis of NRTs. (A) Simulated SERS intensity maps of an Au NRT containing an AuBP core excited at 532, 633, 785, and 830 nm. The 532 nm simulation was performed with a transversal excitation while the 633, 785, and 830 nm with a longitudinal excitation. (B) Experimental SERS area recorded at 1617 cm^–1^ peak of MG for the different laser lines.

In the SERS study, Malachite Green (MG) was used as a Raman probe, which can be encapsulated in the interior void space of the AuAg NRTs during their synthesis as previously reported.^22^ After the preparation, the AuAg NRTs doped with MG (MG-doped AuAg NRTs) were subjected to several washing cycles to ensure the removal of remaining molecules on their outer surface (refer to the experimental part for further details). The resulting MG-doped AuAg NRTs exhibit an aspect ratio of 6.7 with the main LSPR modes at around 1350, 750, and 540 nm (Figure S19 in SI). These modes cannot perfectly match the laser lines (LLs) typically employed in SERS (532, 633, 785, and 830 nm). Nevertheless, FDTD simulations indicated that these LLs are suitable to excite an Au NRT of similar dimensions, generating uniform and intense SERS enhancements inside the NRT as illustrated in Figure 6A (further details in Figure S20B in the SI). The simulated SERS field (|E|^4^/|E_0_|^4^) distribution showed the most intense fields in the inner Au core for the 830, 785, and 633 nm wavelengths upon longitudinal excitation, while for the 532 nm, the transverse excitation shows a homogeneous distribution of the SERS field in the void space.

Then, we performed the experimental SERS analysis of MG-doped AuAg NRTs with four LLs (532, 633, 785, and 830 nm), revealing the presence of the characteristic SERS peaks of MG in all cases (Figure S20C in the SI), as predicted by the FDTD calculations. Figure 6B shows the area analysis of the MG characteristic 1617 cm^–1^ peak for different laser lines. The results indicate the ability to enhance the Raman signal of MG, suggesting the presence of internal hybrid modes in the AuAg NRTs. Notably, a significantly higher intensity was observed for the 633 nm laser line compared to the other laser lines, as the MG molecule is resonant with the laser excitation. Similar SERS intensity values were obtained for 532 and 785 nm laser lines. While this might initially appear to be inconsistent with the simulated maps shown in Figure 6A, it is important to consider that at 532 nm, the SERS field is homogeneous distributed in the void space of the NRT, whereas at 785 or 830 nm, the SERS field is localized close to the Au bipyramid core.^22^

To further confirm this, AuAg NRTs were synthesized under identical conditions but in the absence of MG (Figure S21 in the SI) showing a similar roughness as MG-doped AuAg NRTs. The resulting nanoparticles exhibited similar optical properties, with the main LSPR modes at around 1550 nm, (Figure S20A in the SI). These nanostructures were subsequently functionalized with Malachite Green isothiocyanate (MGi), hereafter referred to as MGi-coated AuAg NRTs. This Raman-active molecule was selected since it binds strongly to the Au surface via the isothiocyanate group.^26^ Besides, it should be noted that the MGi cannot diffuse into the internal void space as the nanostructure is fully enclosed. Furthermore, the SERS ability of MGi-coated AuAg NRTs was analyzed using the four excitation LLs (Figure S20D in the SI). In all cases, a significantly lower signal was observed compared to MG-doped AuAg NRTs (Figure S20E in the SI). This can be attributed to the significantly lower SERS field on the outer particle surface when the NPs were excited with the LLs.

The fact that these AuAg NRTs exhibit tunable optical properties in the NIR region makes them interesting for SEIRA spectroscopy. To investigate their performance for SEIRA, AuAg NRTs with an aspect ratio of 11.6 and a dipolar longitudinal LSPR centered at 2500 nm were chosen (Figure S22 in the SI). The CTAB was selected as the target molecule, characterized by vibrational modes: the −CH_2_– symmetric (2848 cm^–1^) and asymmetric (2918 cm^–1^) stretching modes (Figure S23A in the SI).^12^ The SEIRA spectra of CTAB (10^–4^ M) were acquired in the presence of different concentrations of AuAg NRTs ranging from 10^–9^ M and 10^–14^ M. The results showed that the SEIRA signal increased with increasing NP concentration (specially above 10^–11^ M), demonstrating the capability of the AuAg NRTs for SEIRA (Figure S23A-B in the SI). The SEIRA enhancement factor (EF) was estimated for the different NP concentrations using the equation:
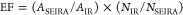
1where *A*_SEIRA_ and *A*_IR_ represent the SEIRA and IR area of the characteristic CTAB peak (2875–2935 cm^–1^) in the presence or absence of AuAg NRTs, respectively. *N*_SEIRA_ and *N*_IR_ are the CTAB concentrations employed for IR and SEIRA characterization, respectively, which in this case is the same. Plotting the SEIRA EF as a function of the AuAg NRTs concentration (Figure S23C in the SI) showed that the highest SEIRA EF, around 10^2^, was achieved at the highest concentration analyzed. It should be noted that this EF could potentially be improved by further increasing the AuAg NRTs concentration and by tuning the dipole LSPR toward the mid-IR to match the absorption band of CTAB at around 3450 nm. Unfortunately, it is beyond the detection limit of our equipment and cannot be confirmed by the narrowed plasmon bands.

## Conclusions

Inspired by theoretical simulations showing the possibility of tuning the LSPR toward NIR with hollow nanorods, we have successfully developed a synthetic protocol for the fabrication of AuAg NRTs with a tailored and narrowed LSPR in the NIR region. The aspect ratio of the Ag PTW sacrificial templates and the amount of gold salt are the two main parameters determining the plasmonic properties of the resulting NRTs. Their extensive electron microscopy characterization confirmed the maintenance of the pentatwinned structure and the presence of residual Ag. Interestingly, experiments performed under oxidative conditions demonstrated the high stability of the AuAg NRTs in comparison with the Ag PTWs.

The in-depth analysis of the optical response of AuAg NRTs, through absorption spectroscopy and EELS, complemented by FDTD simulations, indicated a complex interplay of plasmon modes localized on the outer surface as well as within the void space. Moreover, to shed light on the internal modes, we encapsulated MG within the void space, showcasing significant enhancement in their Raman signals with all the laser lines, indicative of the presence of internal hybrid modes as FDTD simulations predicted. Additionally, we demonstrated the potential of AuAg NRTs for SEIRA, demonstrating a significant enhancement of the IR signals of CTAB as a target molecule was observed.

Consequently, we have elucidated the foundational principles governing the optical properties of these nanostructures. These valuable insights will be useful for the development of next-generation nanomaterials, offering tailored functionalities and enhanced performance in various technological applications.

## Experimental Section

### Materials

Silver nitrate (AgNO_3_, ≥99%), l-ascorbic acid (AA, ≥99%), cetyltrimethylammonium chloride solution (CTAC, 25 wt % in H_2_O), citric acid (C_6_H_8_O_7_, ≥99.5%), sodium borohydride (NaBH_4_, ≥99%) and Malachite Green oxalate salt (MG), were purchased from Sigma-Aldrich. Hydrogen tetrachloroaurate (III) trihydrate (HAuCl_4_·3H_2_O), Malachite Green isothiocyanate (MGi), and cetyltrimethylammonium bromide (CTAB, ≥99%) were obtained from Alfa Aesar, Thermo Fisher, and Across Organics, respectively. All chemicals were employed without further purification. Water was employed as solvent after being purified in a Milli-Q system (Millipore).

### Finite Difference Time Domain (FDTD) Simulations

FDTD simulations of nanoparticles were carried out using Ansys Lumerical Photonics Simulation & Design Software. Perfectly matched layers (PML) were used as boundary conditions of the simulation area, with a total-field scattered-field (TFSF) linearly polarized light as source. Due to the complex anisotropy of the nanoparticles, we have considered eight different polarizations for the incident light. Optical power box monitors were selected to estimate absorption and scattering cross sections, while frequency domain field profile monitors were used to obtain electromagnetic field spatial distributions.

### Synthesis of Pentatwinned Gold Nanorods (Au PTW)

Pentatwinned gold nanorods were synthesized using a seed-growth mechanism previously reported procedure with minor modifications.^20^ In a 20 mL vial, 10 mL of an aqueous solution containing 0.25 mM HAuCl_4_, 0.05 M CTAC, and 5 mM citric acid were prepared. Subsequently, 0.25 mL of 25 mM NaBH_4_ was added under vigorous stirring to obtain gold seeds. After 2 min, the vial was sealed, and the solution was heated at 80 °C for 30 min.

For the growth of the nanoparticles, a solution of 500 mL was prepared, containing 1.25 × 10^–4^ M HAuCl_4_ and 8 mM CTAB. The growth solution was placed in a thermostatic bath at 20 °C. Next, 1.25 mL of 100 mM ascorbic acid was added to the growth solution, resulting in a color change from orange to clear. Subsequently, 5 mL of the seed solution was added to the mixture. After thorough homogenization, the solution was left to react for 12 h at 20 °C.

Upon completion of the synthesis, the solution was centrifuged twice at 9000 rpm for 5 min each, using 10 mL of 0.1 M CTAB as the centrifugation medium. The supernatant was discarded, and the pellet was redispersed in 4 mL of water. To purify the pentatwinned gold nanorods, a specific volume of 25% CTAC was added, resulting in a final concentration of 0.32 M, which induced flocculation of the nanorods due to depletion forces. Finally, the supernatant was removed, and the precipitate was redispersed in 15 mL of 10 mM CTAB.

### Synthesis of the Gold Pentatwinned Bipyramid (Au BP)

Pentatwinned gold bipyramids were synthesized following a previously reported synthetic protocol with minor modifications.^20^ In a 100 mL Erlenmeyer flask, 35 mL of a 50 mM CTAC solution was added, followed by the addition of 400 μL of 25 mM HAuCl_4_. The mixture was gently stirred and kept in a water bath at 30 °C for 10 min. Subsequently, 4 mL of a 50 mM citric acid solution was added, and the solution was maintained at 30 °C for 30 min. Next, 1 mL of freshly prepared 25 mM NaBH_4_ solution was added under vigorous stirring and kept for at least 30 s. The resulting seed solution was aged at 40 °C for a week.

To initiate the growth process, 2 mL of the initial seed solution was added to a 250 mL Erlenmeyer flask containing 200 mL of a solution composed of 100 mM CTAB, 100 mM AgNO_3_, 50 mM HAuCl_4_, and 20 mM HCl. Before adding the seed, 1600 μL of 100 mM ascorbic acid was introduced to the solution at 30 °C, and manual mixing was performed for 5 s. The seed was then added to the solution, and the final mixture was kept at 30 °C for 4 h.

The resulting nanoparticles were initially purified by centrifugation twice at 8000 rpm for 20 min. To further purify the bipyramids and separate them from other morphologies, a flocculation process was employed. The pellet obtained after centrifugation was redispersed in a 350 mM BDAC solution and kept undisturbed at 30 °C overnight. The resultant supernatant was discarded, leaving the bipyramids in the pellet. The pellet was then redispersed in a 1 mM CTAC solution and subjected to three additional rounds of centrifugation (8000 rpm for 20 min each time), with the pellet being redispersed in 1 mM CTAC after each centrifugation step.

### Synthesis of AuBP/AuPTW@Ag PTW Nanoparticles

Silver overgrowth over Au nanoparticles was carried out using a previously reported methodology from Sánchez-Iglesias et al.^23^ To 10 mL of Au PTW or Au BP in water (0.12 mM in terms of Au^0^ and 10 mM CTAC), different volumes of AgNO_3_ (10 mM) and ascorbic acid (100 mM) were added under vigorous stirring at 60 °C and allowed to react for 1 h. It is important to note that the molar ratio [AA]:[Ag^+^] was maintained constant at a value of 4, and the maximum volume of AgNO_3_ added in each step was 0.5 mL. After the reaction, the resulting solution was centrifuged and redispersed in 10 mL of CTAB 10 mM. For AuPTWs as seed, the Ag^+^/Au^0^ ratios were 2.8, 3.8, 5.7, and 7.1. For AuBPs as seeds, the Ag^+^/Au^0^ ratios were 4.1, 7.0, 9.1, 12.5, 14.7 and 18.9.

### Synthesis of AuAg Nanorattles

The synthesis of nanorattles was carried out following a previously reported procedure with some modifications.^27^ Briefly, 0.2 mL of 0.2 M ascorbic acid was added to 5 mL of AuBP/AuPTW@Ag PTW in water. Subsequently, a certain volume of 0.5 mM HAuCl_4_ was added under stirring, using a syringe pump at a flow rate of 25 μL/min. The added HAuCl_4_ volume depended on the Ag^+^/Au^0^ molar ratio and the type of seed used. For AuPTW@Ag PTW, the added volume of HAuCl_4_ was 6 mL for Ag^+^/Au^0^ ratios of 2.8, 3.8, and 5.7, and 10 mL for an Ag^+^/Au^0^ ratio of 7.1. In the case of AuBP@Ag PTW, 5 mL of HAuCl_4_ was added for Ag^+^/Au^0^ ratios of 4.1 and 7.0, while 10 mL was added for ratios of 12.5. For higher Ag^+^/Au^0^ ratios, 16 mL of HAuCl_4_ was added for ratios of 14.7 and 18.9. After synthesis, the nanoparticles were washed through centrifugation cycles and redispersed in CTAB 10 mM.

For the SERS study, AuBP@Ag PTW was prepared using the same procedure as described above with an Ag^+^/Au^0^ ratio of 9.05. To form the AuAg NRTs, 0.2 mL of 0.2 M ascorbic acid were added to a solution of 5 mL of AuBP@Ag PTW followed by the addition of 22 mL of 0.5 mM HAuCl_4_ under stirring with a syringe pump at a flow rate of 25 μL/min. For the encapsulation of Malachite Green molecules, 0.4 mL of a 10^–2^ M solution was added prior to the pump-driven addition. To functionalize the outer surface of the AuAg NRTs, 0.4 mL of a 10^–2^ M solution of Malachite Green isothiocyanate was added under vigorous stirring and left to stir at room temperature overnight. In both cases, the nanoparticles were washed twice by centrifugation.

For the SEIRA study, AuBP@Ag PTWs were prepared with an Ag^+^/Au^0^ ratio of 13.1. To obtain the nanorattles, 10 mL of AuBP@Ag PTWs were utilized, followed by the addition of 0.4 mL of 0.2 M ascorbic acid and 30 mL of 0.5 mM HAuCl_4_, added using a pump with a flow rate of 25 μL/min.

### Study of the Stability of Nanoparticles in the Presence of an Oxidizing Agent

Hydrogen peroxide solution, a strong oxidizing agent, was used to study the stability of nanoparticles following the method of Mayer et al.^28^ with minor modifications. A volume of 1 mL of NPs was centrifuged twice and redispersed in 2 mL of 1 mM CTAC. In the case of AuBP@Ag PTW, 6 μL of H_2_O_2_ 35% w/w (0.1% final concentration) was added to the solution and study stability following the kinetic evolution of their optical properties. In the case of AuBP@AuAg NRTs, a 10-fold greater hydrogen peroxide concentration was used (60 μL of H_2_O_2_ 35% w/w, 1% final concentration) to study the stability of NPs.

### Characterization Methods

UV–Vis–NIR absorption spectra of aqueous colloidal solutions were recorded using a Cary 8454 (Agilent) or a Cary 5000 (Agilent) spectrophotometer. Quartz cuvettes with different optical path lengths (0.1, 0.5, and 1 cm) were used. Transmission electron microscopy (TEM) images were obtained with a JEOL JEM 1010 transmission electron microscope operating at an acceleration voltage of 100 kV. Scanning electron microscopy (SEM) characterization was performed using a JEOL JSM-6700F FEG scanning electron microscope operating at an acceleration voltage of 15 kV in secondary-electron imaging (SEI) mode. High-angle annular dark-field scanning transmission electron microscopy (HAADF-STEM) images and energy-dispersive X-ray spectroscopy (EDS) maps were obtained using a “cubed” aberration-corrected Thermo Fisher Scientific Titan microscope equipped with a SuperX EDS system operating at 300 kV. A Fischione model 2020 single tilt holder was used for tomography tilt series acquisition within a tilting range from −70 to +70°. The tilt increment was set to be 2° for HAADF-STEM tomography using a fast acquisition methodology in which the focusing and tracking steps are performed while the holder continuously tilts, reducing the acquisition time and therefore mitigating any electron beam damage to the sample.^29^ In the context of EDS tomography, the same aforementioned tomography holder was employed. Here, the acquisition of EDS maps was extended to a minimum duration of 15 min, ensuring the acquisition of a sufficient number of counts at intervals of 10° from −70 to +70°. Both tilt series were aligned using a phase correlation implemented in the ASTRA Toolbox for MATLAB.^30,31^ EELS experiments were performed on a Thermo Fisher Scientific Titan microscope operating at 120 kV. The monochromator was excited to achieve an energy resolution of 0.1, as measured from the full width at half-maximum of acquired zero-loss peaks.

### Surface-Enhanced Raman Scattering (SERS)

For the SERS characterization, 2 mL of the colloidal sample was put in a quartz 1 cm cuvette and put in a liquid holder for its characterization. The SERS measurements were performed using a Renishaw InVia Reflex system. The spectrograph used a high-resolution grating (1200 or 1800 grooves/cm) with additional band-pass filter optics, a confocal microscope, and a 2D-CCD camera. Laser excitation was carried out at 532 nm (22.3 mW), 633 nm (5.65 mW), 785 nm (51.6 mW), and 833 nm (1.06 mW) with an acquisition time of 10 s and 5 accumulations. All the SERS spectra were recorded 10 times and an average was performed. All the spectra were acquired and treated using WiRE Software v. 4.3 (Renishaw, UK).

### Surface-Enhanced Infrared Absorption (SEIRA)

The IR characterization was performed on a FTIR Nicolet 6700 Continuum Infrared microscope spectrometer (Thermo Scientific). To perform the IR characterization a drop of 200 μL of 0.1 M CTAB was dropped on a spherical substrate of MgF_2_. The drop was let dry for 1 h on a desiccator connected to a mechanical vacuum pump. After that the substrate was introduced in the IR system and a transmittance spectrum was recorded. To avoid the background effect of the substrate, an IR spectrum of the MgF_2_ substrate was recorded and substrate. Finally, the transmission was transformed into extinction. The same protocol was followed to characterize the SEIRA spectra of the nanoparticles, a drop of 200 μL of the nanoparticles with the desired concentration and containing 0.1 mM CTAB was dried under vacuum on a MgF_2_ substrate.

## Data Availability

The data that support the findings of this study are available at the open-access repository Zenodo.org, DOI:10.5281/zenodo.11238562

## Supporting Information Available

FDTD simulations of the extinction response, electric field distributions of the plasmonic nanostructures (MP4)
Additional UV–Vis-NIR, TEM, HAADF-STEM, EELS characterization of the nanostructures. Time-resolved optical characterization of the galvanic replacement combined with seeded growth of AuBP@Ag PTWs and AuPTW@Ag PTWs. Study of stability of AuBP@Ag PTWs and AuBP@AuAg NRTs against oxidation with H_2_O_2_. SEIRA measurements (PDF)
