# Supplementary material for: Pentatwinned AuAg Nanorattles with Tailored Plasmonic Properties for Near-Infrared Applications
Source: Chem Mater. 2024 Sep 13;36(18):8763–72. doi: 10.1021/acs.chemmater.4c01443 (PMC11428089; doi:10.1021/acs.chemmater.4c01443)
Supplement: Supplementary file 2 — cm4c01443_si_002.pdf [file cm4c01443_si_002.pdf]

Electronic Supporting Information

Pentatwinned AuAg Nanorattles with Tailored Plasmonic Properties for Near Infrared Applications

Daniel García-Lojo[a,b,1], Sergio Rodal Cedeira[a,b,1], Sara Núñez-Sánchez[a,b], Daniel Arenas-Esteban[c], Lakshminarayana Polavarapu[a], Sara Bals[c], Jorge Pérez-Juste[a,b], Isabel Pastoriza-Santos[a,b].

[a] CINBIO, Universidade de Vigo, Departamento de Química Física, Campus Universitario As Lagoas, Marcosende, 36310 Vigo, Spain

[b] Galicia Sur Health Research Institute (IIS Galicia Sur), 36310 Vigo, Spain.

[c] EMAT, University of Antwerp, Groenenborgerlaa 171, 2020 Antwerp, Belgium.

E-mail: pastoriza@uvigo.gal

[1] These authors contributed equally to this work

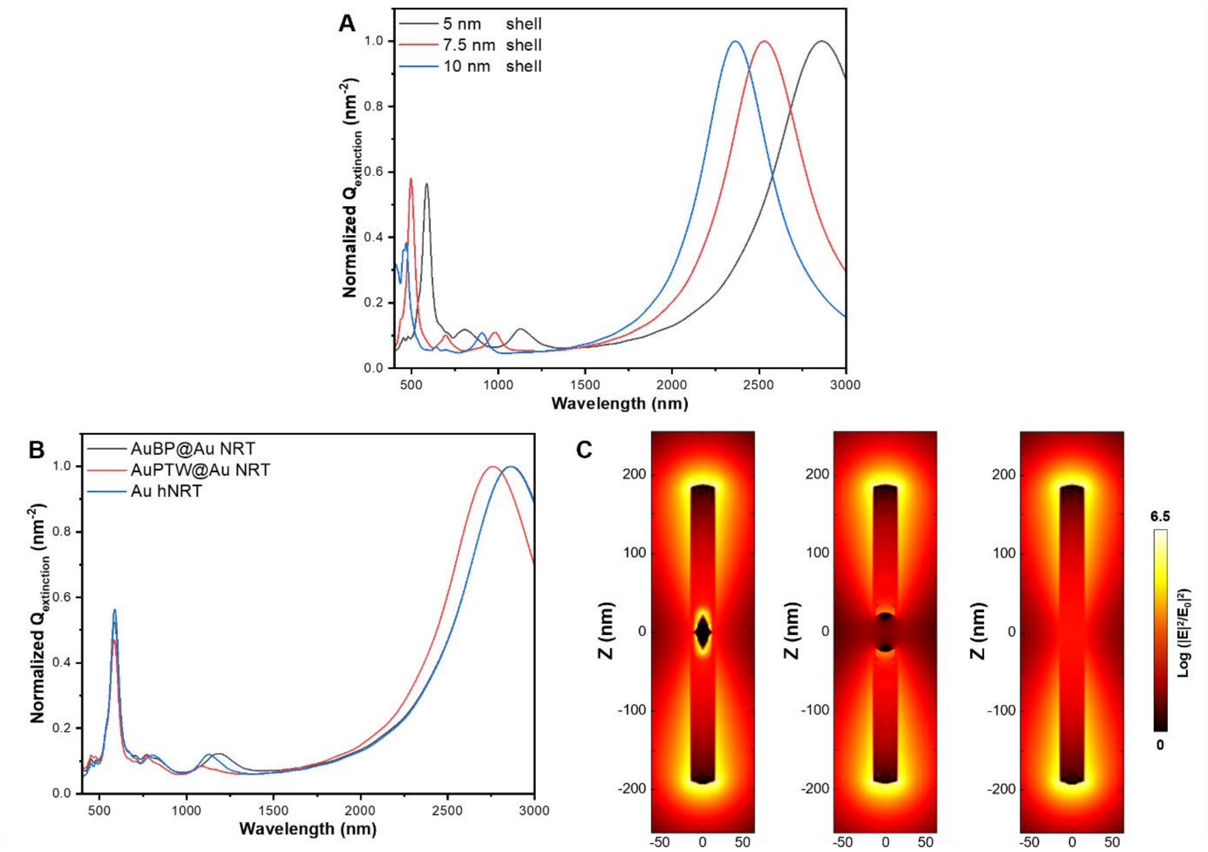

Figure S1. Simulated extinction properties and electric field distribution of Au nanorattles (Au NRTs). (**A**) Normalized finite difference time domain (FDTD) simulations of the extinction response of single hollow pentatwinned Au nanorod (Au hPTW) with different shell thicknesses: 5, 7.5, and 10 nm. (**B**) Calculated extinction spectra of single Au NRT with different cores: gold bipyramid (AuBP@Au NRT), pentatwinned gold nanorod (AuPTW@Au NRT), and without any core (Au hPTW). (**C**) FDTD simulations of the electric field distribution of AuBP@Au NRT (left), AuPTW@Au NRT (middle), and Au hPTW (right). In all cases, the nanoparticles were illuminated at the longitudinal LSPR wavelength with a parallel polarization with respect to the NRT long-axis.

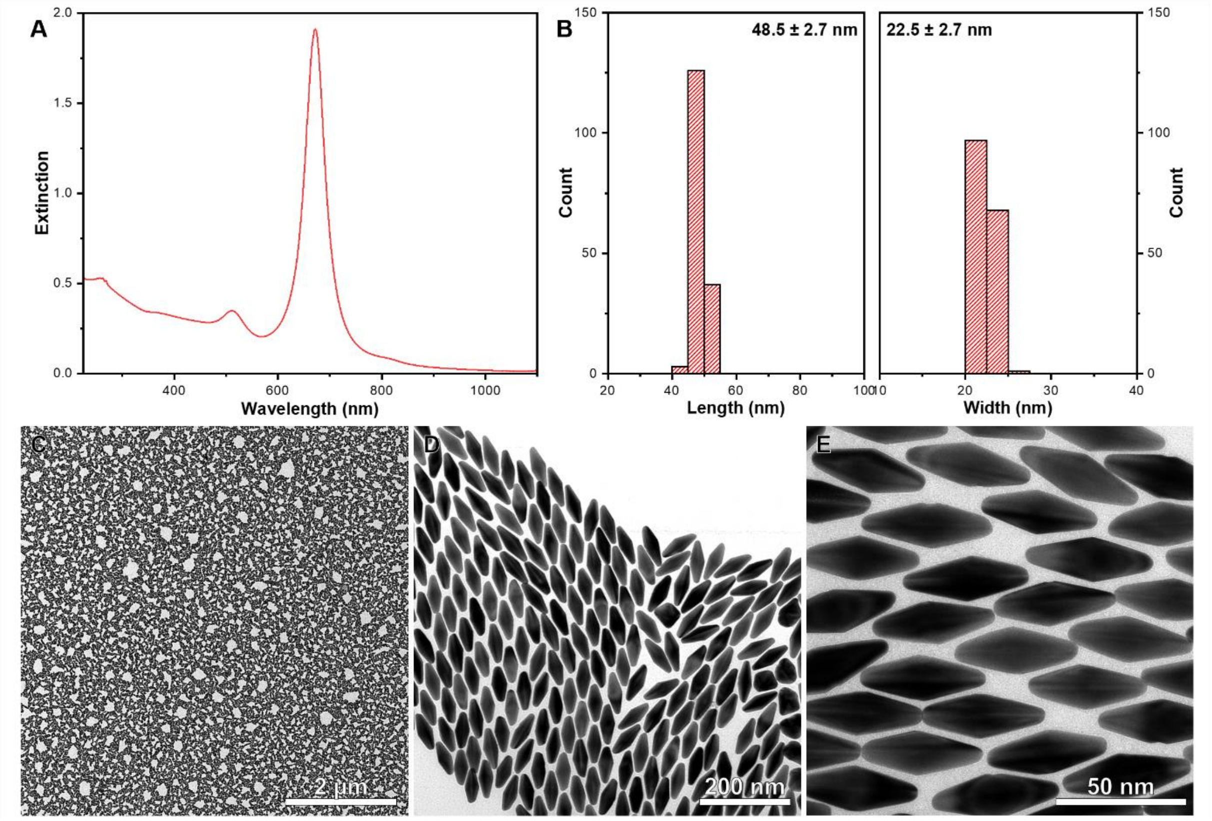

Figure S2. Characterization of pentatwinned gold bipyramids (Au BPs). (**A**) UV-vis-NIR extinction spectrum of the Au BPs in water. (**B**) Histograms obtained from the analysis of TEM images. (**C-E**) TEM images of Au BPs at different magnifications.

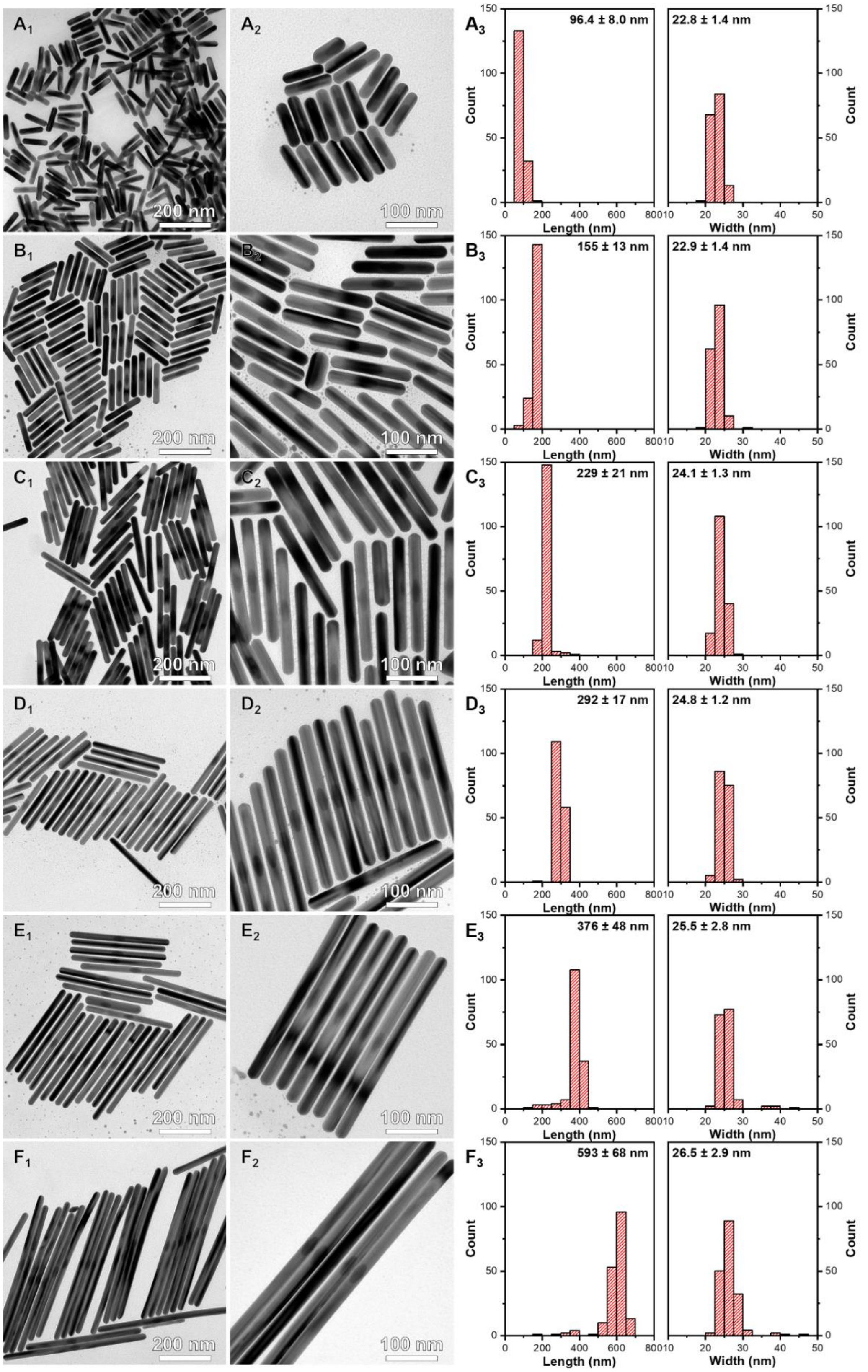

Figure S3. Characterization of pentatwinned silver nanoparticles containing an Au BP core (AuBP@Ag PTWs). (**A-F**) TEM images of different aspect ratio AuBP@Ag PTW nanostructures at different magnifications (left) and histogram of the length and width sizes of the corresponding nanoparticles (right). The nanostructures have an aspect ratio of 4.2 (**A**), 6.8 (**B**), 9.5 (**C**), 11.8 (**D**), 15.0 (**E**), and 22.7 (**F**).

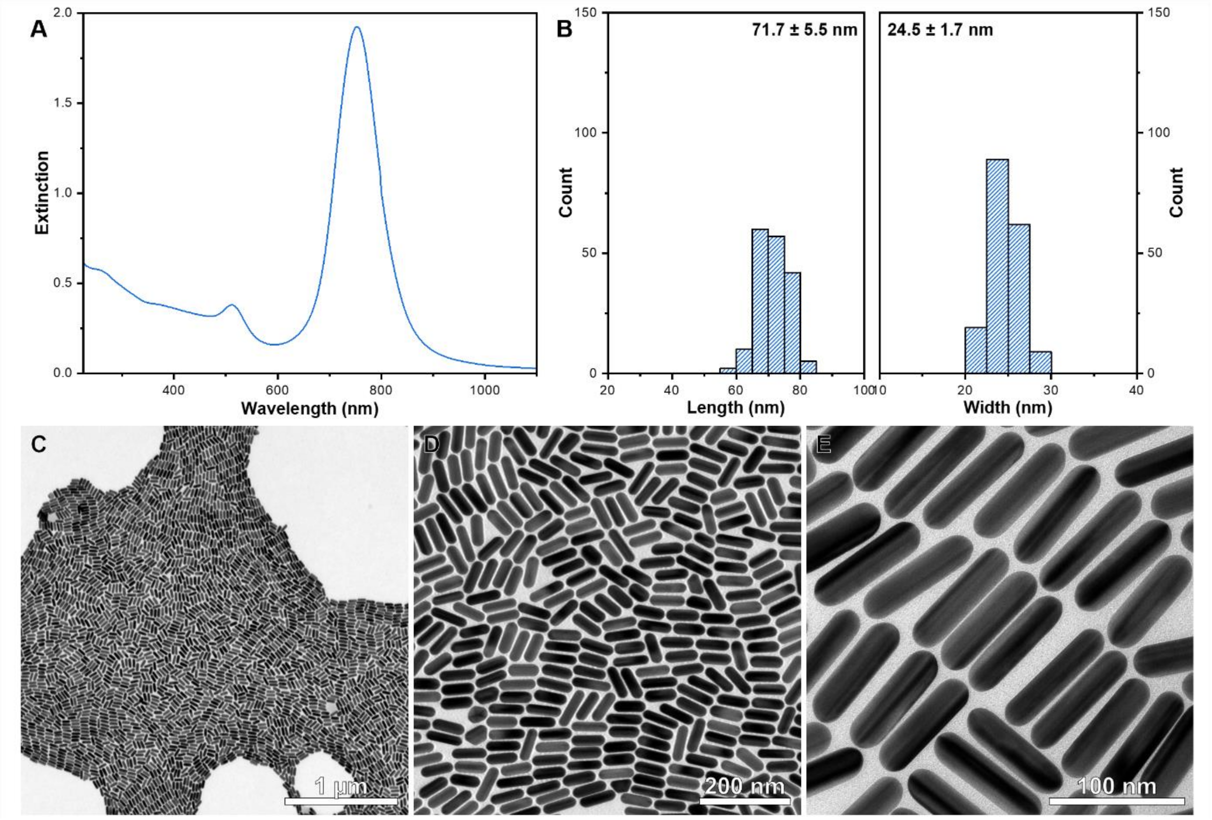

Figure S4. **Characterization of pentatwinned gold nanorods (Au PTWs).** (**A**) UV-vis-NIR extinction spectrum of the Au PTWs in water. (**B**) Histogram obtained from the analysis of TEM images. (**C-E**) TEM images of Au PTWs at different magnifications.

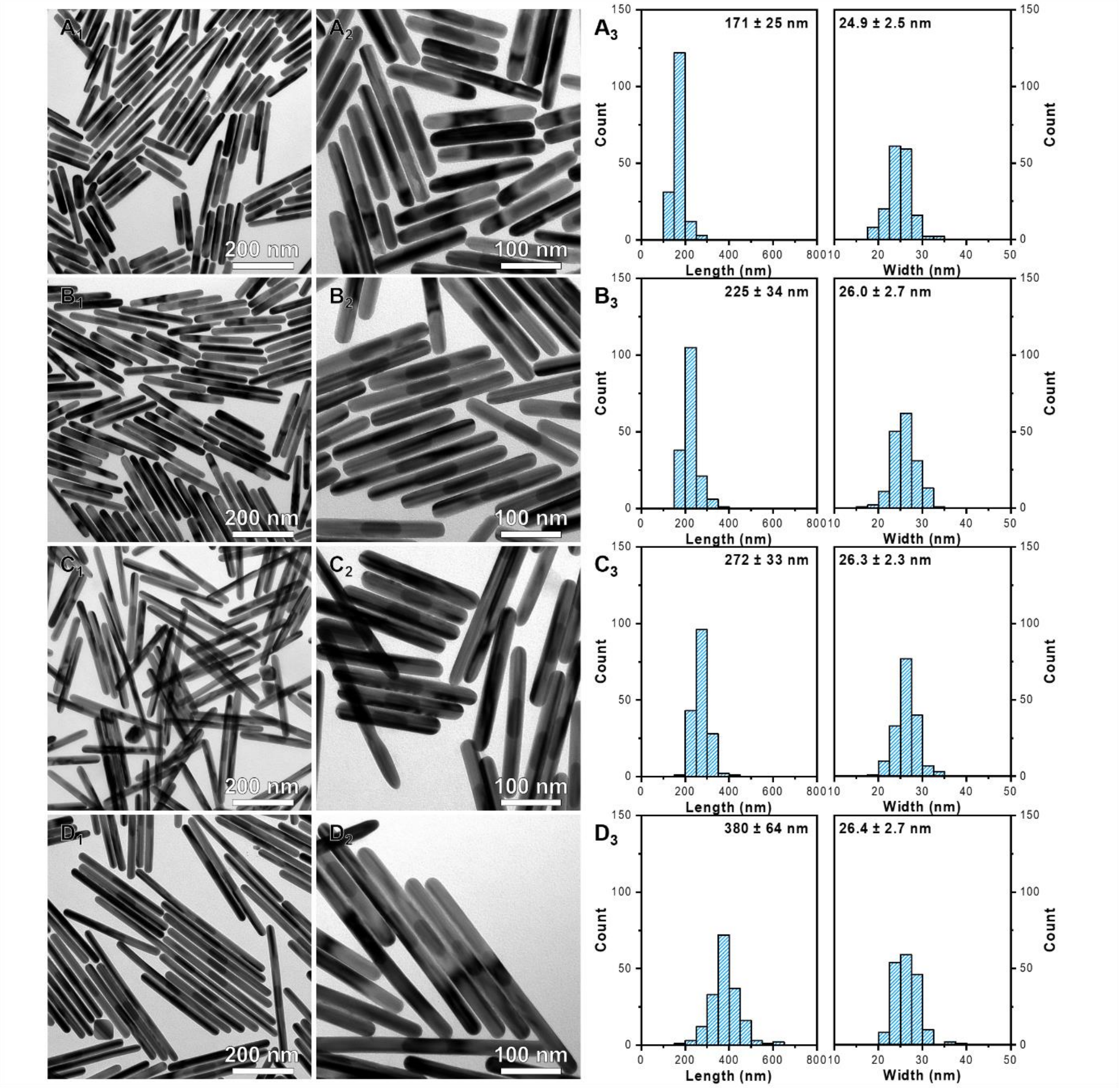

Figure S5. Characterization of pentatwinned silver nanoparticles containing an Au PTW core (AuPTW@Ag PTWs). (**A-F**) TEM images of different aspect ratio AuPTW@Ag PTWs at various magnifications (left) and corresponding histograms of the length and width (right). The AuPTW@Ag PTWs have aspect ratios of 7.0 (**A**), 8.8 (**B**), 10.4 (**C**), and 14.6 (**D**).

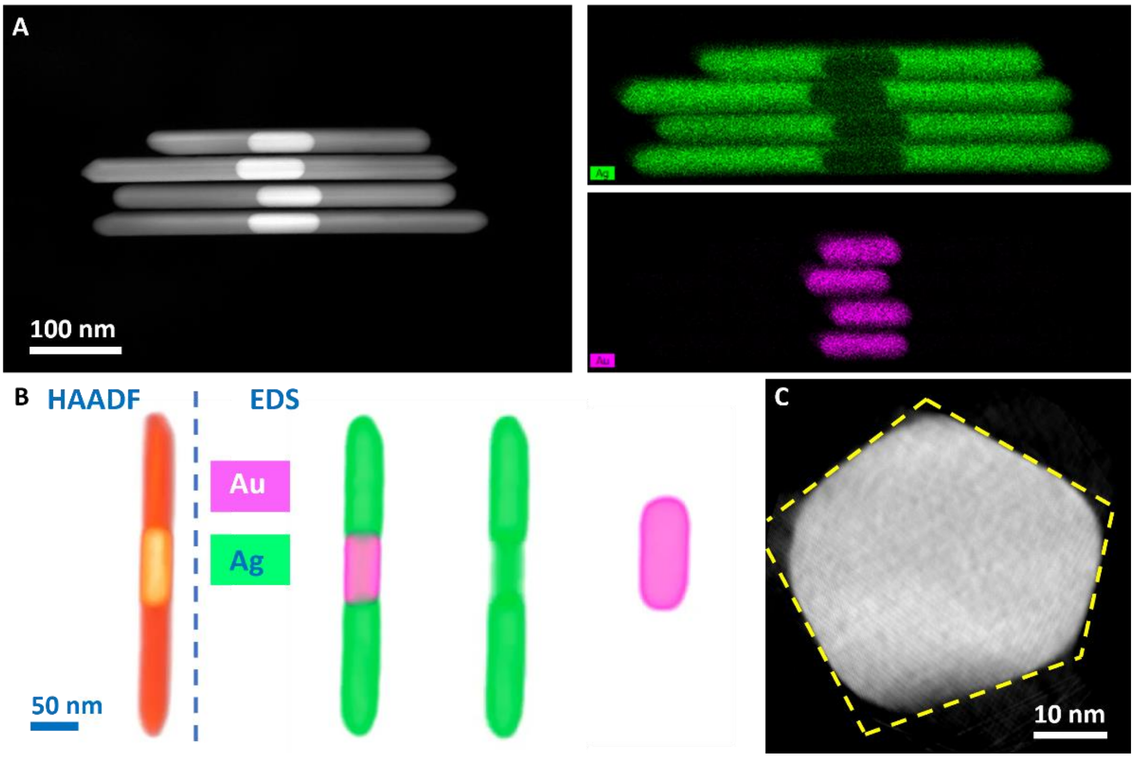

Figure S6. Electronic microscopy characterization of AuPTW@Ag PTWs. (**A**) HAADF-STEM image of AuPTW@Ag PTWs and their corresponding EDS maps where the Au and Ag elemental distribution is shown. (**B**) HAADF-STEM (orange) and EDS (pink/green) tomography of one selected AuPTW@Ag PTW. (**C**) Orthoslice from a HAADF-STEM tomography where the pentagonal cross section is marked in yellow dashed lines.

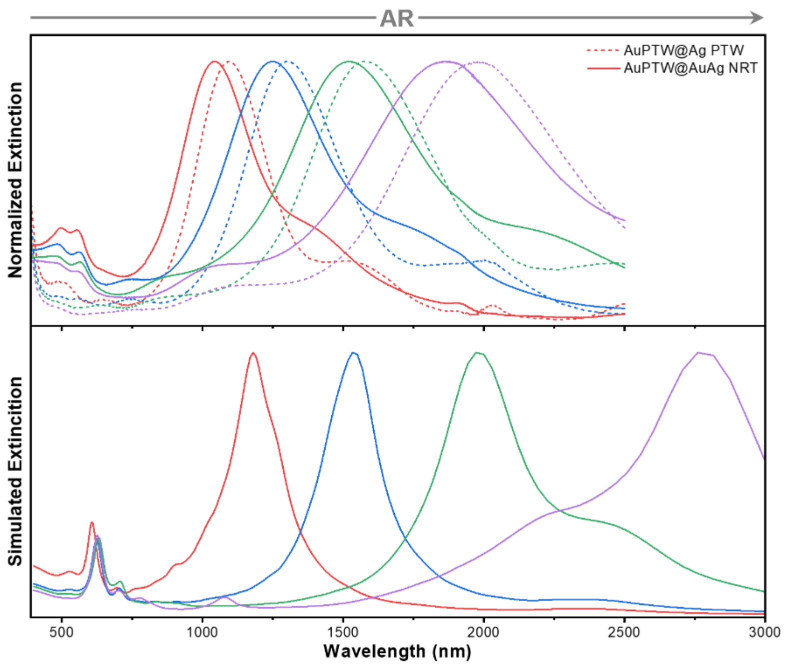

Figure S7. Experimental and simulated extinction spectra of AuAg NRTs with an Au PTW core (AuPTW@AuAg NRTs). Top panel: Normalized extinction spectra of AuPTW@Ag PTWs with different aspect ratios (dotted line) and the corresponding AuPTW@AuAg NRTs (solid line). Bottom panel: corresponding simulated extinction spectra of Au NRTs with an Au PTW core. The aspect ratios of the final AuPTW@AuAg NRTs are 4.5 (red), 6.1 (blue), 7.9 (green), and 9.8 (violet). The simulated extinction spectra were obtained employing a model nanostructure with the measured TEM dimensions for each aspect ratio (see Table S3 on the ESI for further details) and with a shell thickness of 7 nm.

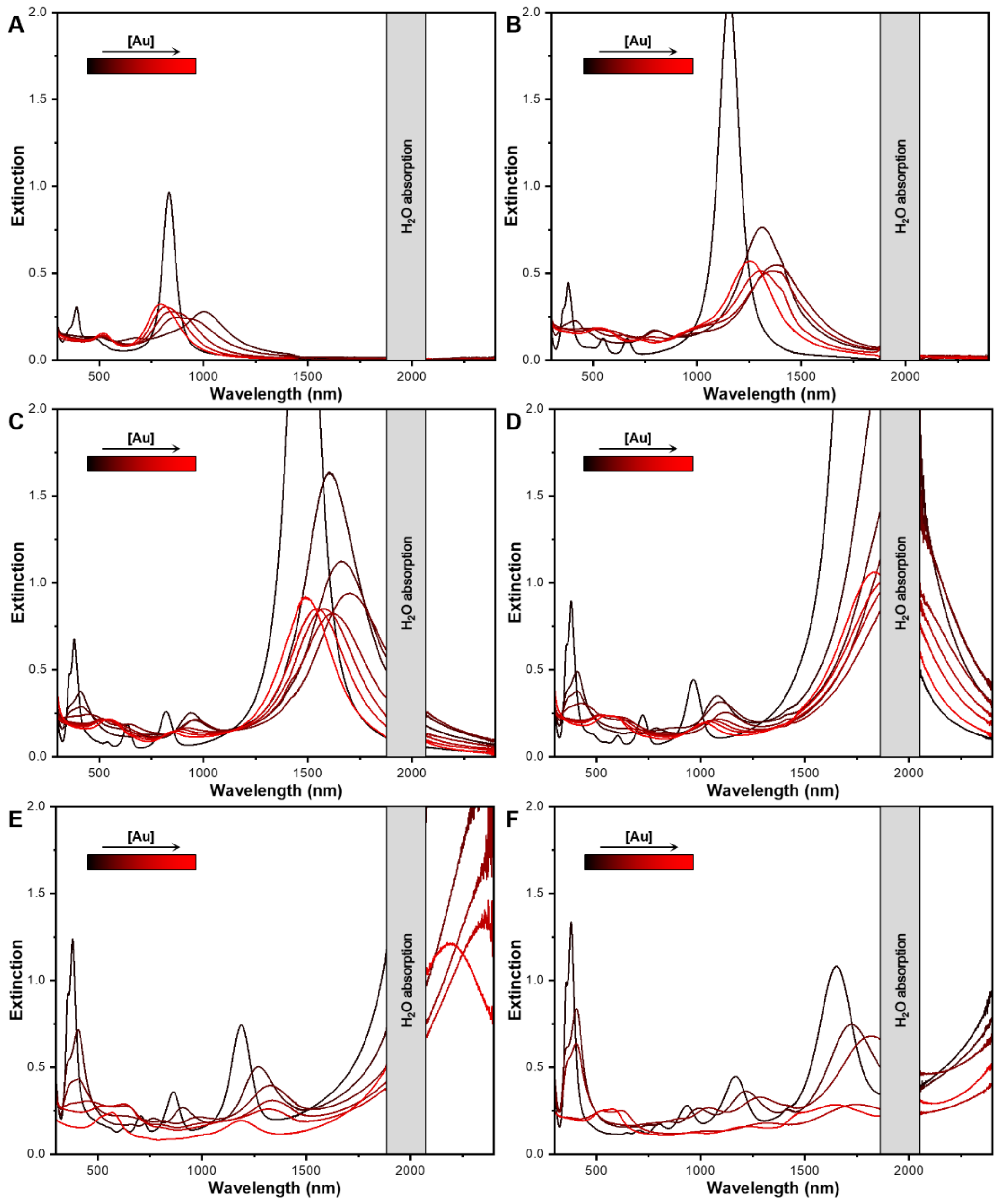

Figure S8. Time-resolved optical characterization of the galvanic replacement combined with seeded growth of AuBP@Ag PTWs. (**A-F**) Evolution of the extinction spectra of AuBP@Ag PTWs with starting aspects ratios of 4.2 (**A**), 6.8 (**B**), 9.5 (**C**), 11.8 (**D**), 15.0 (**E**), and 22.7 (**F**). The H_2O absorption does not allow the analysis between 1800 and 2050 nm.

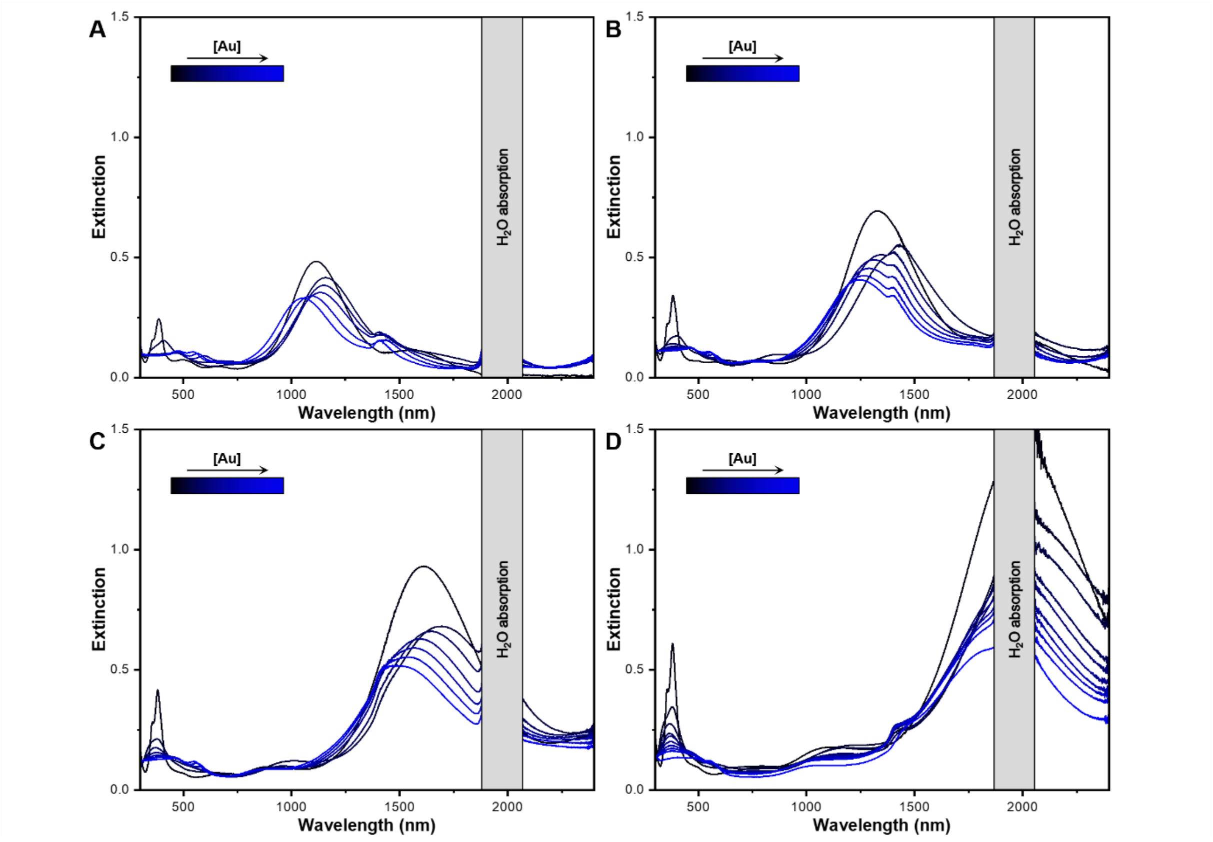

Figure S9. Time-resolved optical characterization of the galvanic replacement combined with seeded growth of AuPTW@Ag PTWs. (**A-D**) Evolution of the extinction spectra of AuPTW@Ag PTWs with starting aspects ratios of 7.0 (**A**), 8.8 (**B**), 10.4 (**C**), and 14.6. The H_2O absorption does not allow the analysis between 1800 and 2050 nm.

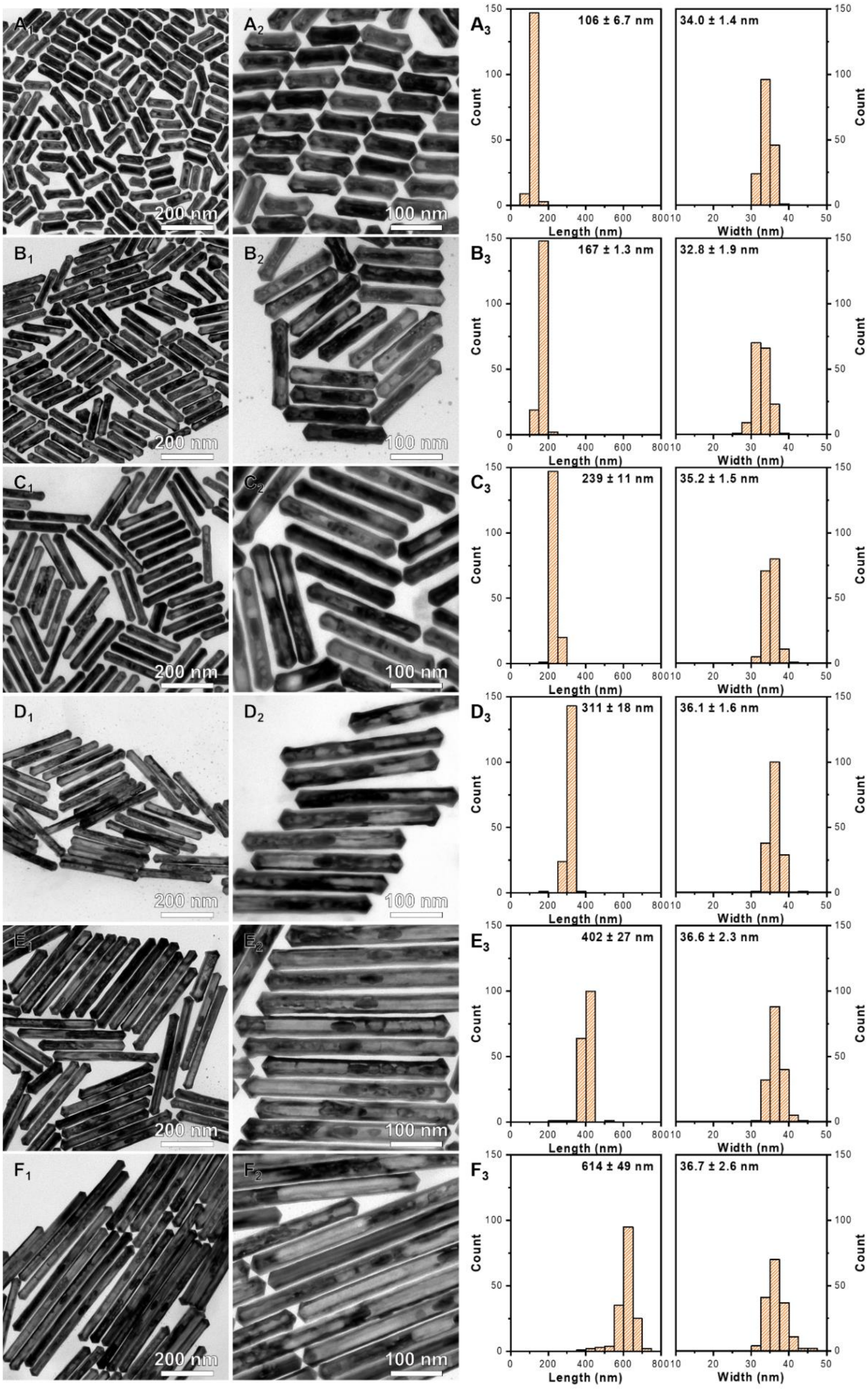

Figure S10. Characterization of NRTs obtained from AuBP@Ag PTWs. (**A-F**) TEM images of different aspect ratio NRTs at different magnifications (left) and corresponding histograms, length and width (right). The nanostructures have an aspect ratio of 3.1 (**A**), 5.1 (**B**), 6.8 (**C**), 8.6 (**D**), 11.0 (**E**), and 16.9 (**F**).

Table S1. Summary of AuBP@Ag PTW (left) and NRT (right) dimensions and dipolar LSPR wavelengths. The values and their standard deviation were obtained from TEM images of Figures S3 and S10. The aspect ratio value refers to the ratio length/width of each of the nanoparticle batches measured.

Fig	LSPR (nm)	Length (nm)	Width (nm)	Aspect Ratio	Fig	LSPR (nm)	Length (nm)	Width (nm)	Aspect Ratio	Shell Thickness (nm)
**S3A**	836	96.4 ± 8.0	22.8 ± 1.4	4.2 ± 0.4	**S10A**	785	106 ± 6.7	34.0 ± 1.4	3.1 ± 0.3	5.6 ± 1.0
**S3B**	1160	155 ± 13	22.9 ± 1.4	6.8 ± 0.7	**S10B**	1232	167 ± 10	32.8 ± 1.9	5.1 ± 0.4	4.95 ± 1.2
**S3C**	1494	229 ± 21	24.1 ± 1.3	9.5 ± 0.9	**S10C**	1489	239 ± 11	35.2 ± 1.5	6.8 ± 0.4	5.55 ± 1.0
**S3D**	1750	292 ± 17	24.8 ± 1.2	11.8 ± 0.9	**S10D**	1817	310 ± 18	36.1 ± 1.6	8.6 ± 0.6	5.65 ± 1.0
**S3E**	2185	376 ± 48	25.5 ± 2.8	15.0 ± 2.4	**S10E**	2204	402 ± 48	36.6 ± 2.3	11.0 ± 1.0	5.55 ± 1.8
**S3F**	(*)	593 ± 68	26.5 ± 2.9	22.7 ± 3.4	**S10F**	(*)	614 ± 68	36.7 ± 2.6	16.9 ± 1.9	5.50 ± 1.8

(*) The dipole LSPR was red-shifted beyond the instrument's detection limit. The shell thicknes was estimated by the difference between the average width of the AuAg NRT and the average width of the AuBP@Ag PTW used as templates.

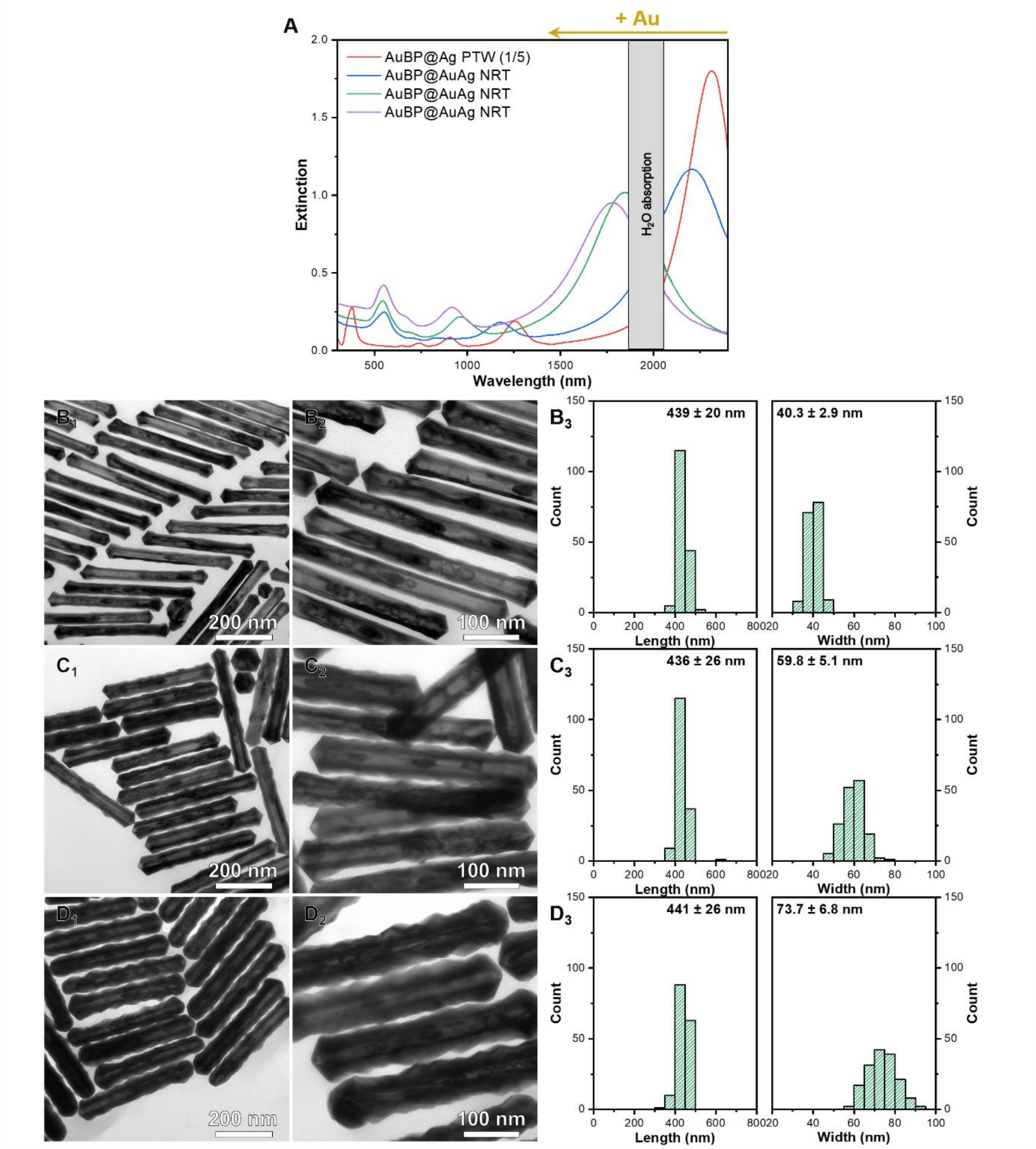

Figure S11. Characterization of NRTs obtained from AuBP@Ag PTWs via the addition of an excess of Au precursor. (**A**) Extinction spectra of NRTs obtained with different Au^{3+}/Au^{0} molar ratio; 26.3 (blue), 36.8 (green), and 61.3 (violet). For clarity, the optical response of the initial AuBP@Ag PTWs was scaled 1/5. The H_2O absorption does not allow the analysis between 1800 and 2050 nm. (**B-D**) Corresponding TEM images at different magnifications (left) and corresponding histograms, length and width, (right) of the NRTs.

Table S2. Summary of dimensions and dipolar LSPR wavelength of NRTs obtained from AuBP@Ag PTWs via the addition of gold precursor excess. The values and their standard deviation were obtained from TEM images of Figure S11. The Aspect Ratio value refers to the ratio length/width of each of the nanoparticle batches measured.

Figure	LSPR (nm)	Length (nm)	Width (nm)	Aspect Ratio
**S11B**	2205	439 ± 20	40.3 ± 2.9	10.9 ± 1.0
**S11C**	1853	436 ± 26	59.8 ± 5.1	7.3 ± 0.8
**S11D**	1778	441 ± 26	73.7 ± 6.8	6.0 ± 0.7

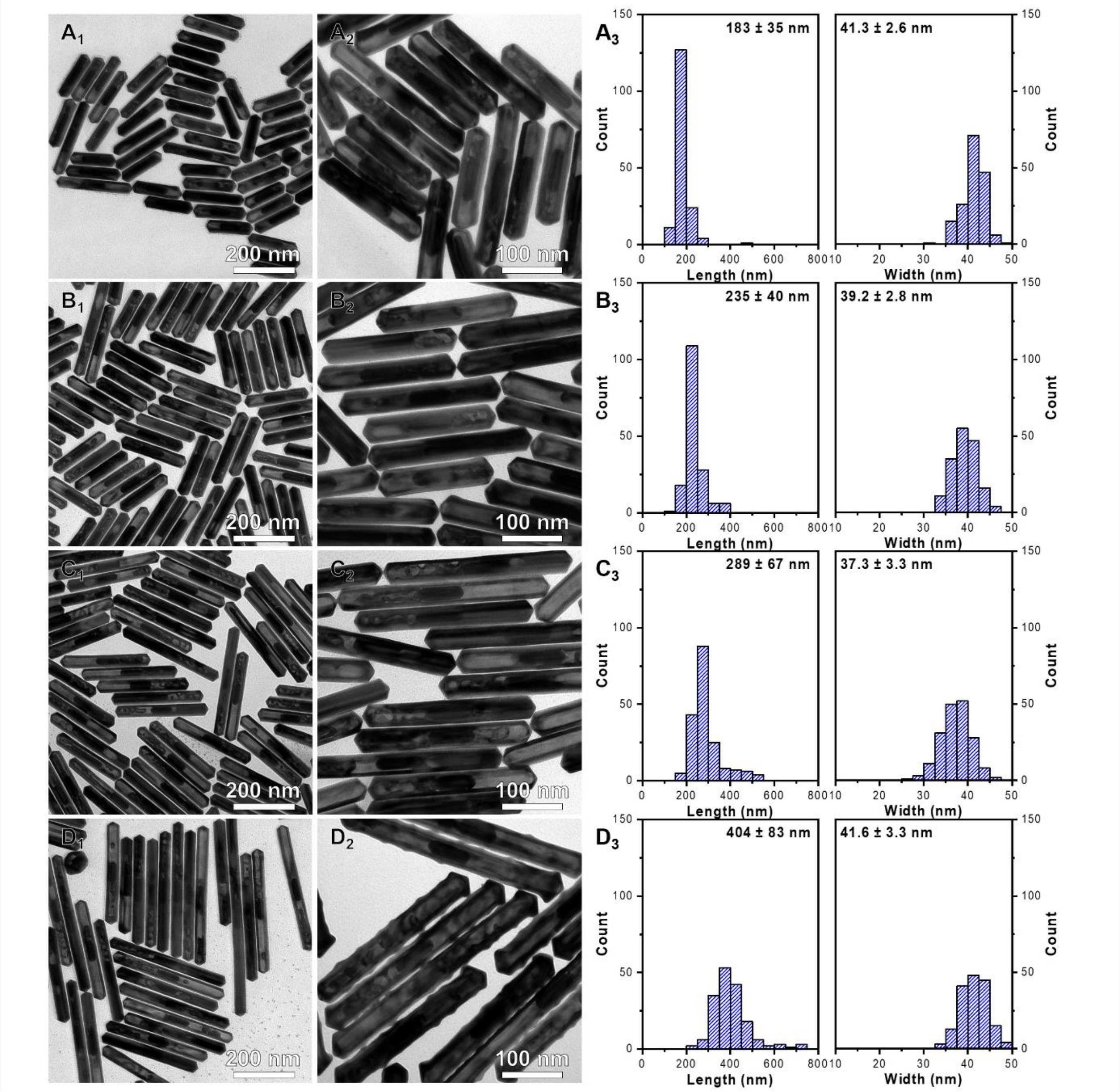

Figure S12. Characterization of NRTs obtained from AuPTW@Ag PTWs. (**A-D**) TEM images at different magnifications (left) and corresponding histograms, length, and width, (right) of NRTs obtained from AuPTW@Ag PTWs with different aspect ratios: 4.5 (**A**), 6.1 (**B**), 7.9 (**C**), and 9.8 (**D**).

Table S3. Summary of dimensions and dipolar LSPR wavelength of AuPTW@Ag PTWs and corresponding NRTs. The values and their standard deviation were obtained from TEM images of Figures S5 and S12. The Aspect Ratio value refers to the ratio length/width of each of the nanoparticle batches measured.

Figure Number	LSPR (nm)	Length (nm)	Width (nm)	Aspect Ratio	Figure Number	LSPR (nm)	Length (nm)	Width (nm)	Aspect Ratio	Shell Thickness (nm)
**S5A**	1094	171 ± 25	24.9 ± 2.5	7.0 ± 1.4	**S12A**	1044	183 ± 35	41.3 ± 2.6	4.5 ± 1.2	8.2 ± 2.1
**S5B**	1302	225 ± 34	26.0 ± 2.7	8.8 ± 2.0	**S12B**	1249	235 ± 40	39.2 ± 2.8	6.1 ± 1.3	6.6 ± 2.3
**S5C**	1578	272 ± 33	26.3 ± 2.3	10.4 ± 1.7	**S12C**	1521	289 ± 67	37.3 ± 3.3	7.9 ± 2.4	5.5 ± 2.8
**S5D**	1978	380 ± 64	26.4 ± 2.7	14.6 ± 3.3	**S12D**	1864	404 ± 83	41.6 ± 3.3	9.8 ± 2.3	7.6 ± 2.7

(*) The shell thicknes was estimated by the difference between the average width of the AuAg NRT and the average width of the AuPTW@Ag PTW used as templates.

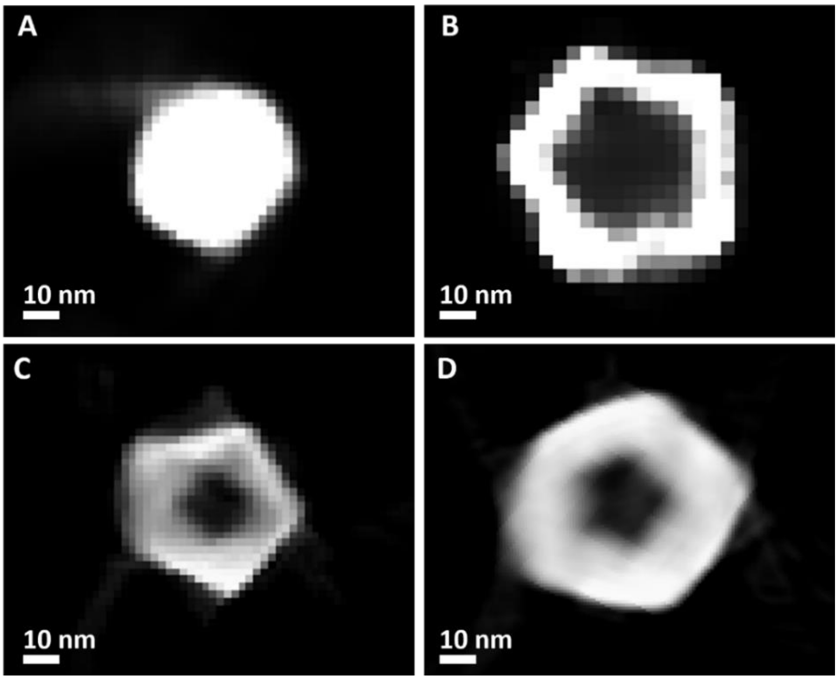

Figure S13. Orthoslices obtained from the 3D HAADF-STEM reconstruction of different nanostructures. (**A**) AuPTW@Ag PTW, (**B**) AuPTW@AuAg NRT, (**C**) AuBP@AuAg NRT with a thin shell, and (**D**) AuBP@AuAg NRT with a thicker shell. All the orthoslices evidence the pentagonal cross section.

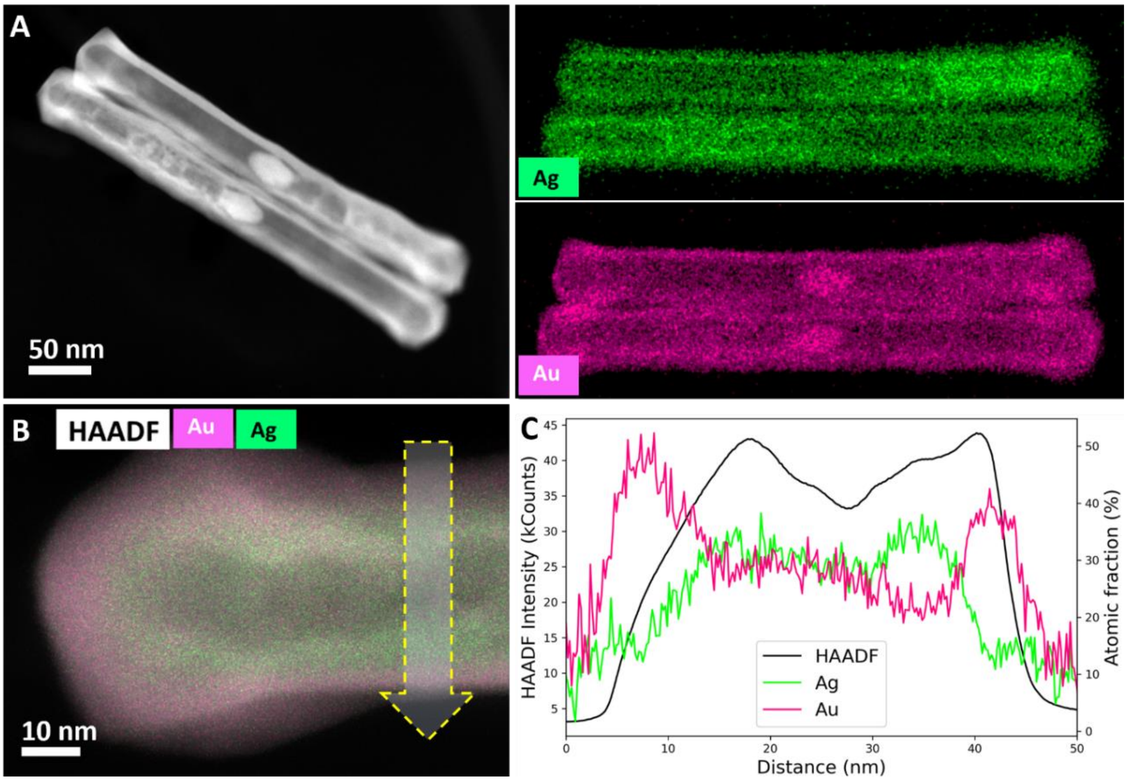

Figure S14. Electronic microscopy characterization of AuBP@AuAg NRTs with a thin shell. (A) HAADF-STEM image of two AuBP@AuAg NRTs and their corresponding EDS map evidencing the presence of Ag. (**B**) Higher magnification EDS map of the AuBP@AuAg NRT tip. (**C**) Line profile performed over the area marked with a dashed yellow arrow in B. The Au (pink) and Ag (green) content is represented in atomic fraction.

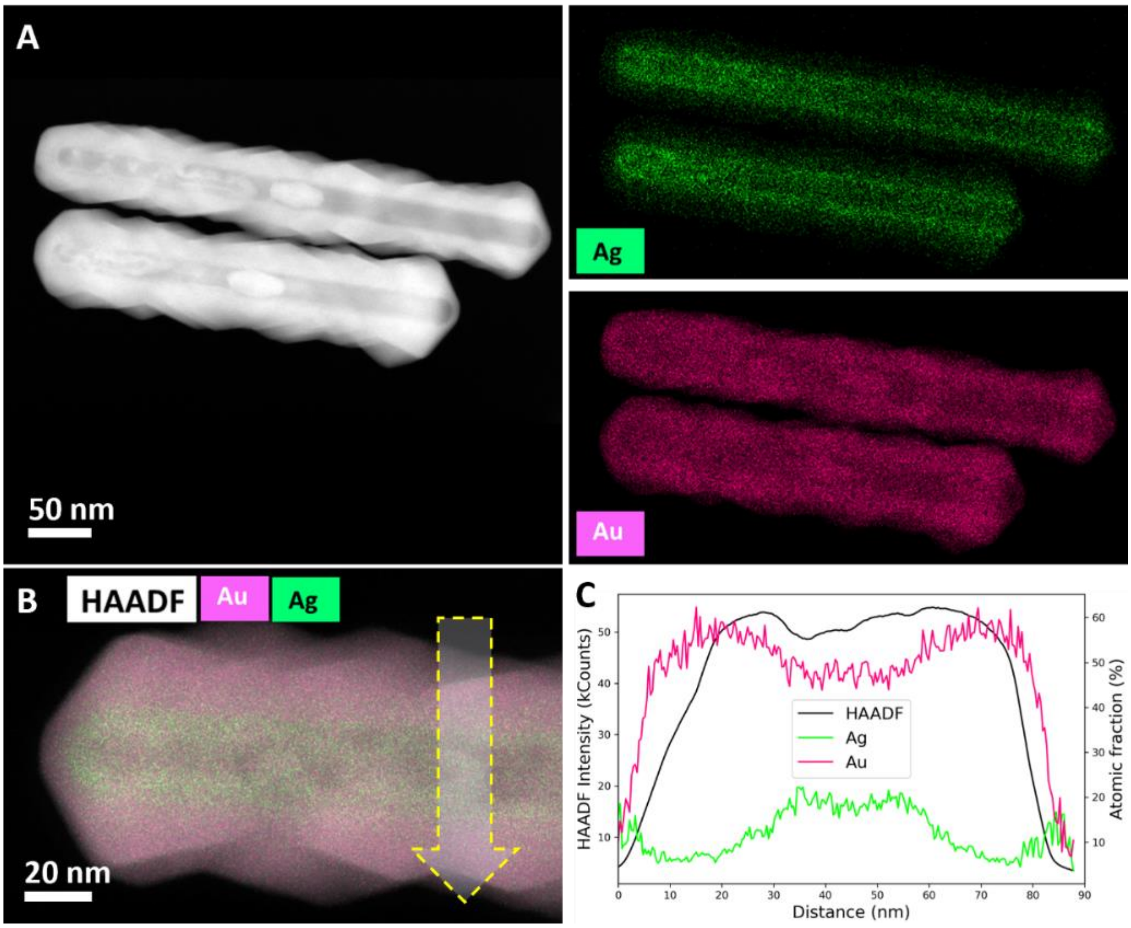

Figure S15. Electronic microscopy characterization of AuBP@AuAg NRTs with a thicker shell. (**A**) HAADF-STEM image of a couple of AuBP@AuAg NRTs with a thicker shell and their corresponding EDS map where Ag can be still detected within the inner part of the Au shell. (**B**) Higher magnification EDS map on the tip of one AuBP@AuAg NRT. (**C**) Line profile performed over the area marked with a dashed yellow arrow. The Au (pink) and Ag (green) content is represented in atomic fraction.

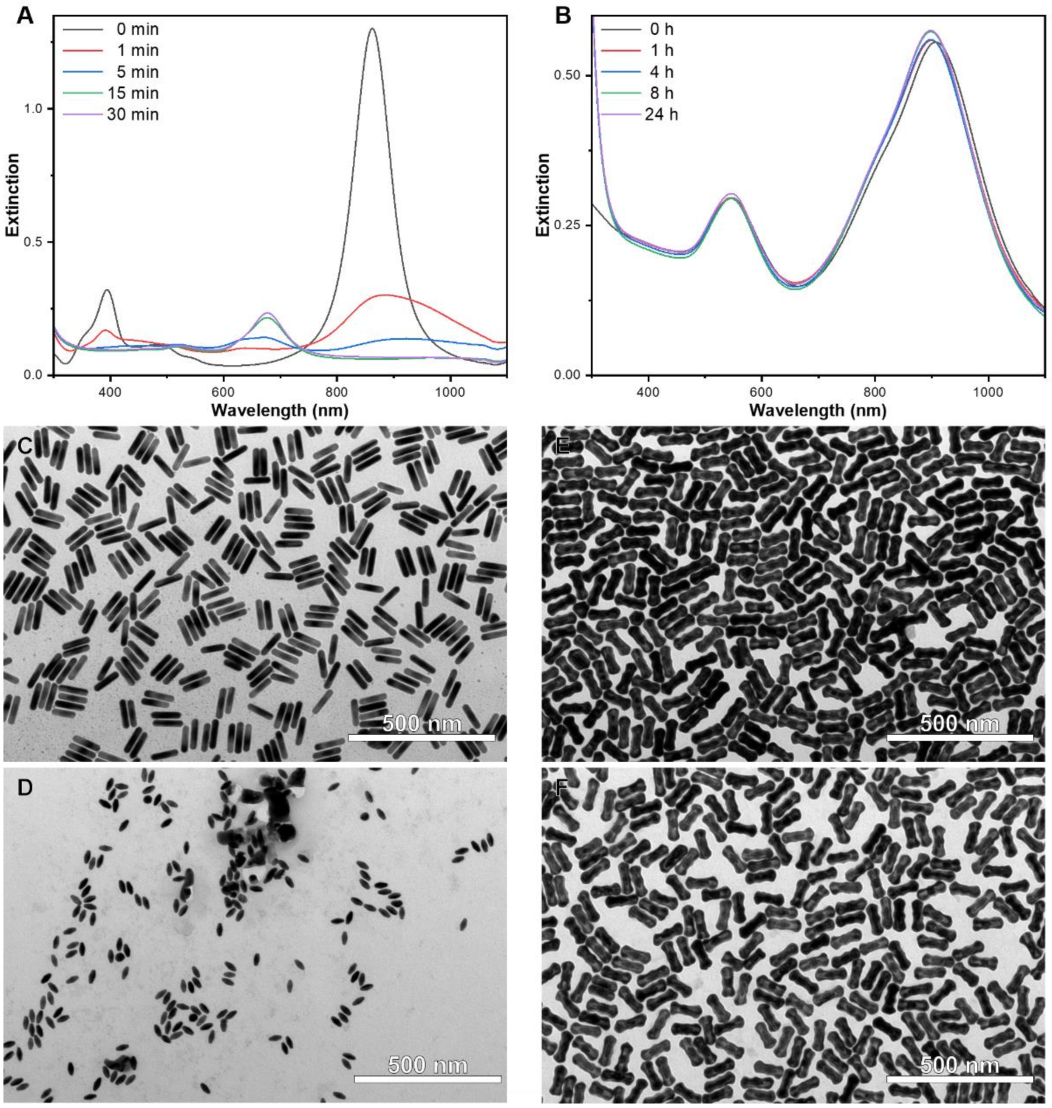

Figure S16. Study of stability of AuBP@Ag PTWs and AuBP@AuAg NRTs against oxidation with H_2O_2. (**A-B**) Time-resolved extinction spectra of colloidal dispersions of AuBP@Ag PTWs (A) and AuBP@AuAg NRTs (B) in the presence of 0.1 and 1% (w/w) of H_2O_2, respectively. (**C-D**) TEM characterization of AuBP@Ag PTWs before (C) and 30 min after (D) the addition of H_2O_2. (E-F) TEM characterization of AuBP@AuAg NRTs before (E) and 24 h after (F) the addition of H_2O_2.

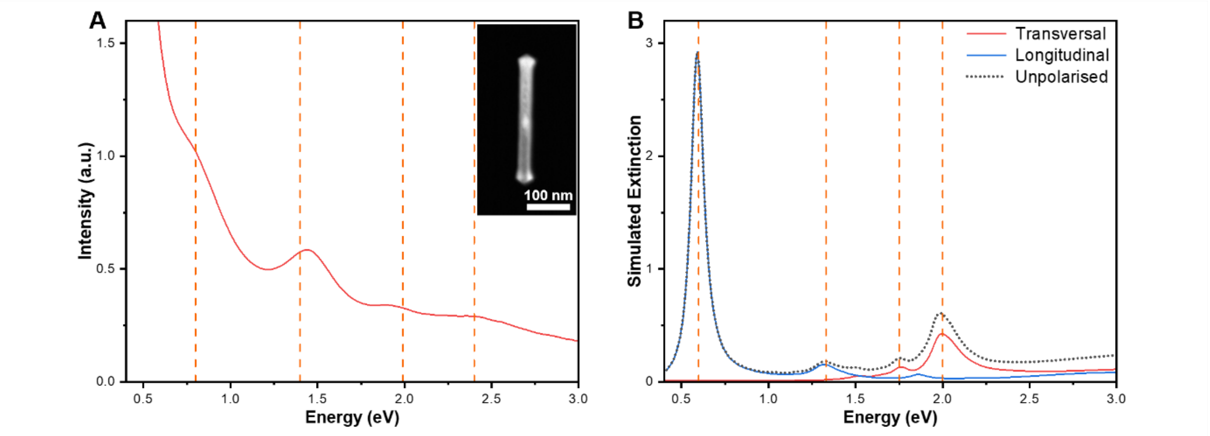

Figure S17. EELS and simulated extinction spectra of AuAg NRTs. (**A**) Average EELS spectrum of a single AuBP@AuAg NRT with an AR of 10. Inset: Corresponding HAADF image. (**B**) FDTD simulated extinction spectra for an AuBP@Au NRT. The different spectra correspond to different excitation conditions (longitudinal, transversal and unpolarized excitation) as indicated. In both graphs, the orange dashed lines represent the excitation energies of the electric field distribution maps of Figure 4A.

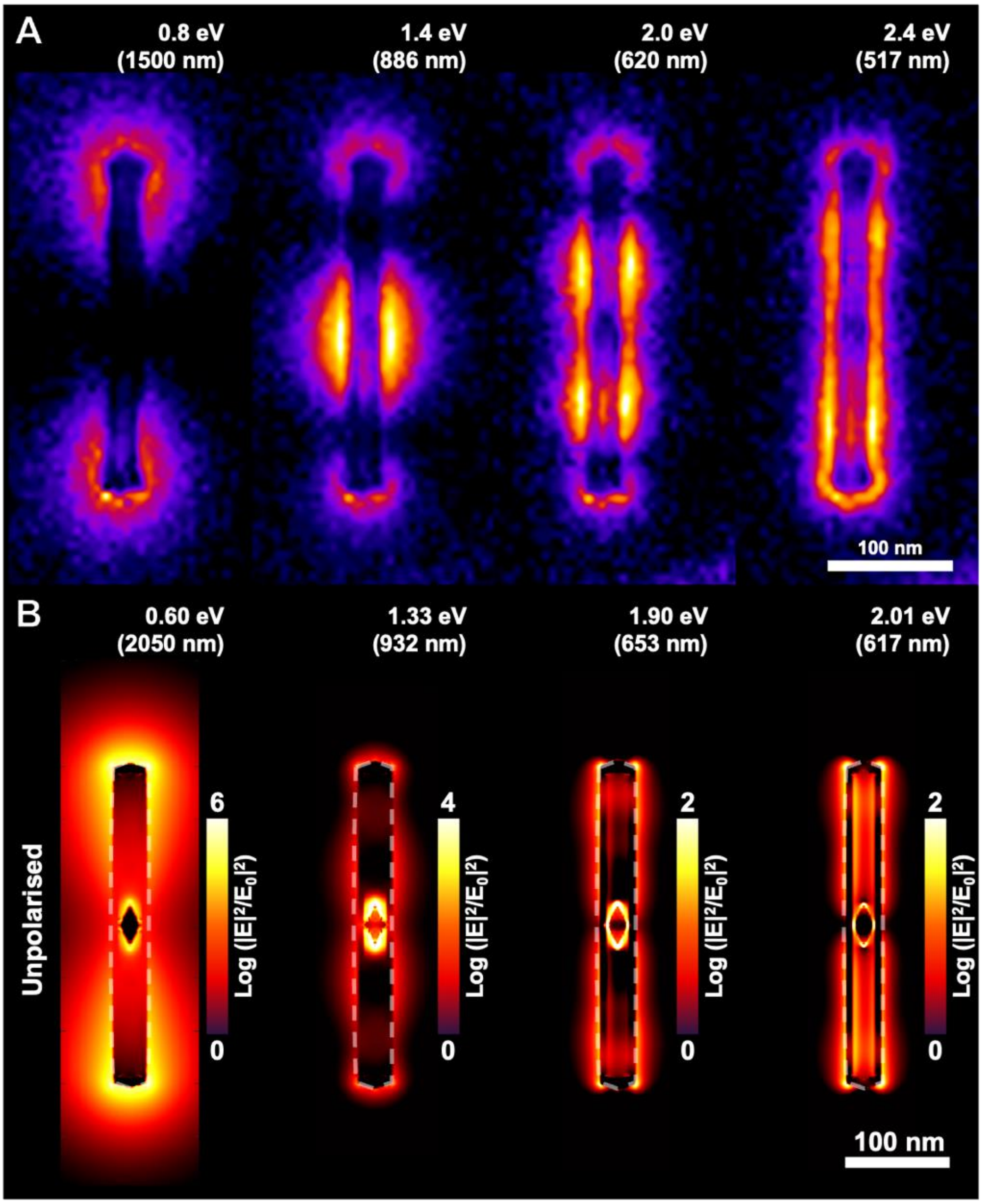

Figure S18. Experimental and FDTD simulated near-field distribution on individual NRTs upon an unpolised excitation with different energies. (**A**) Normalized EELS distribution map acquired after excitation of an AuBP@AuAg NRT at 0.8, 1.4, 2.0, and 2.4 eV, as indicated. The colour scale is represented in the logarithm scale. (**B**) FDTD simulated electric field distribution maps of an AuBP@Au NRT at 0.6, 1.33, 1.90, and 2.01 eV with an unpolarised excitation. The color scale is represented in a logarithm scale and is the same for each energy. The dashed line represents the outer contour of the nanostructure**.**

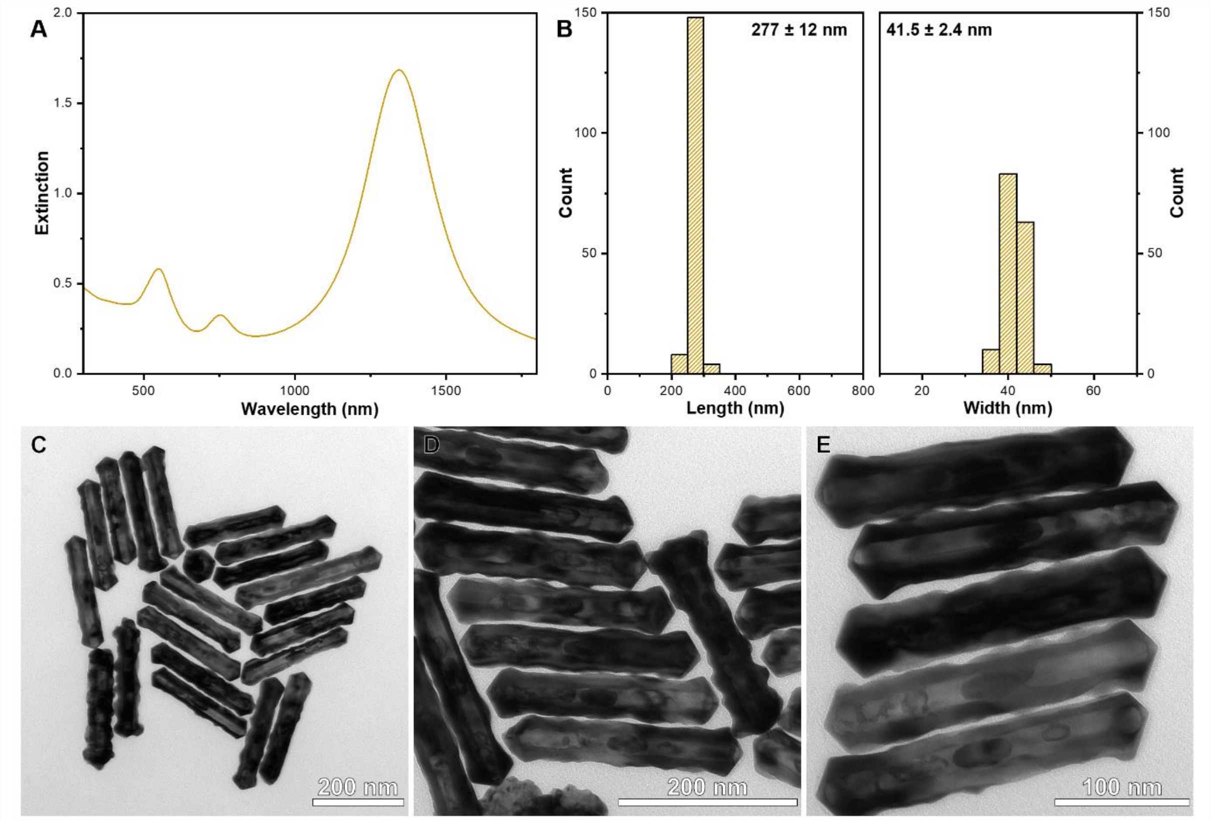

Figure S19. Characterization of malachite green (MG) encapsulated AuAg NRTs (MG-doped AuAg NRT). (**A**) vis-NIR extinction spectrum of the colloids in water. (**B**) Histogram obtained from the analysis of TEM images. (**C-E**) TEM images of MG-doped AuAg NRTs at different magnifications.

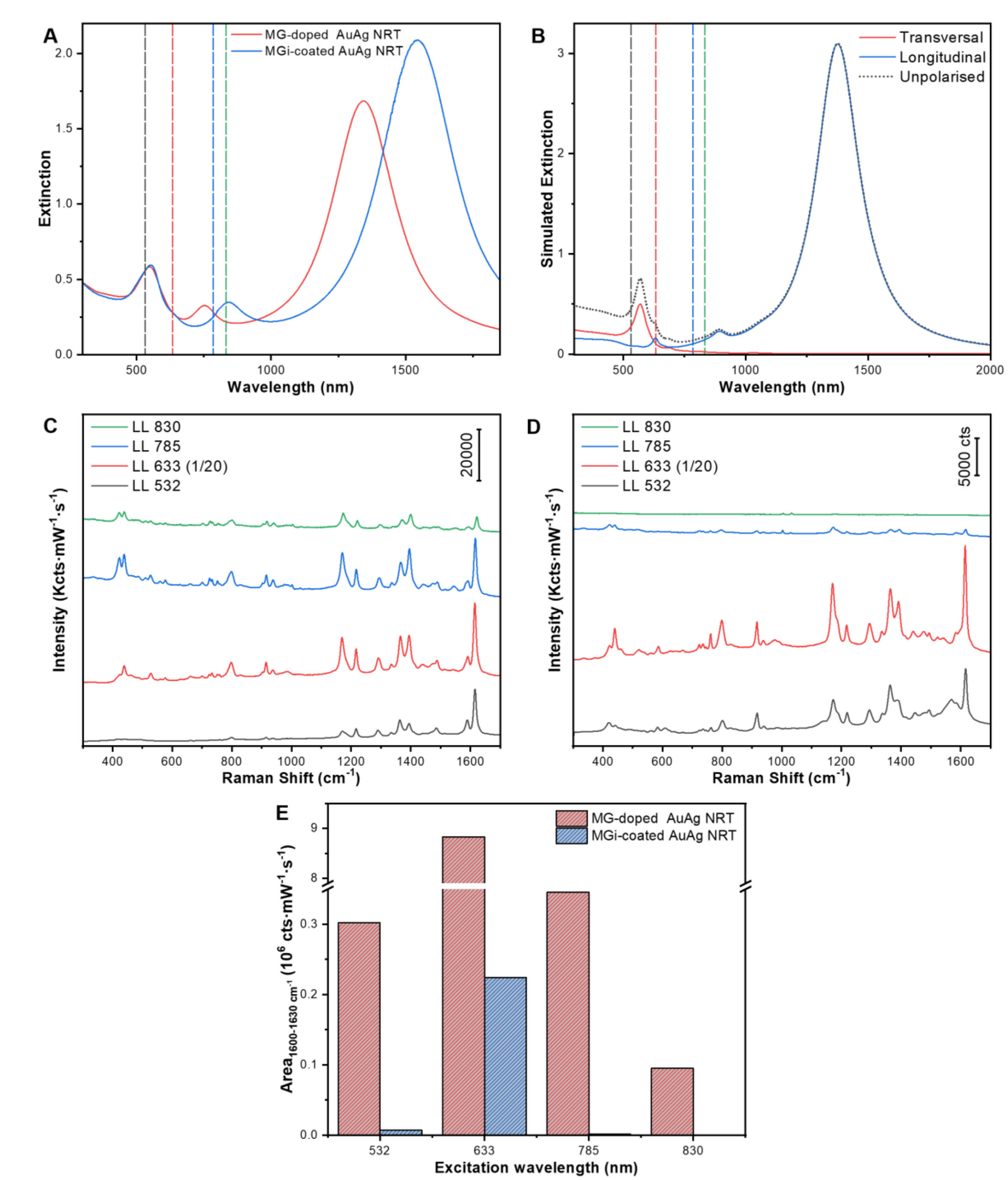

Figure S20. SERS characterization of MG encapsulated AuAg NRTs (MG-doped AuAg NRTs) and AuAg NRTs post-synthetic functionalized with Malachite Green isothiocyanate (MGi-coated AuAg NRT). (**A**) Vis-NIR extinction spectrum of MG-doped AuAg NRT and MGi-coated AuAg NRT in water. (**B**) Simulated extinction spectrum of an Au NRT employed as model for the SERS simulations. In A and B, the vertical dashed lines correspond to the excitation laser lines employed for the theoretical and experimental SERS studies. (**C**) Experimental SERS characterization of a colloidal dispersion of MG-doped AuAg NRT employing different laser lines. (**D**) Experimental SERS characterization of a colloidal dispersion of MGi-coated AuAg NRT employing the different laser lines. (**E**) Histogram that represents the area of the characteristic peak of MG/MGi at 1617 cm^{-1} employing the different laser lines for the MG-doped AuAg NRT and MGi-coated AuAg NRT.

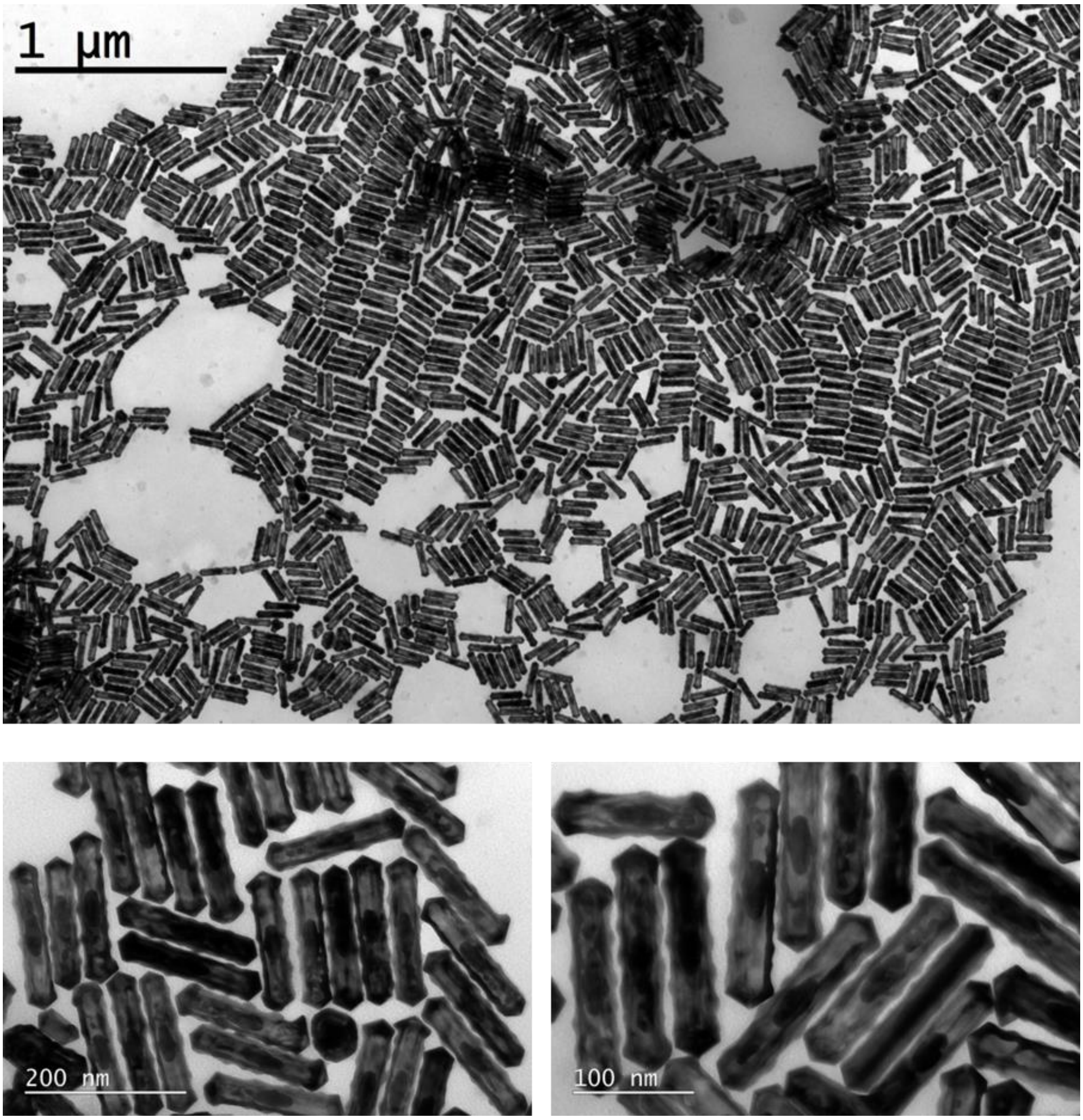

Figure S21. TEM characterization of AuAg NRTs. TEM images of AuAg NRTs synthesized in the absence of MG and post-synthetic functionalized with Malachite Green isothiocyanate (MGi-coated AuAg NRT).

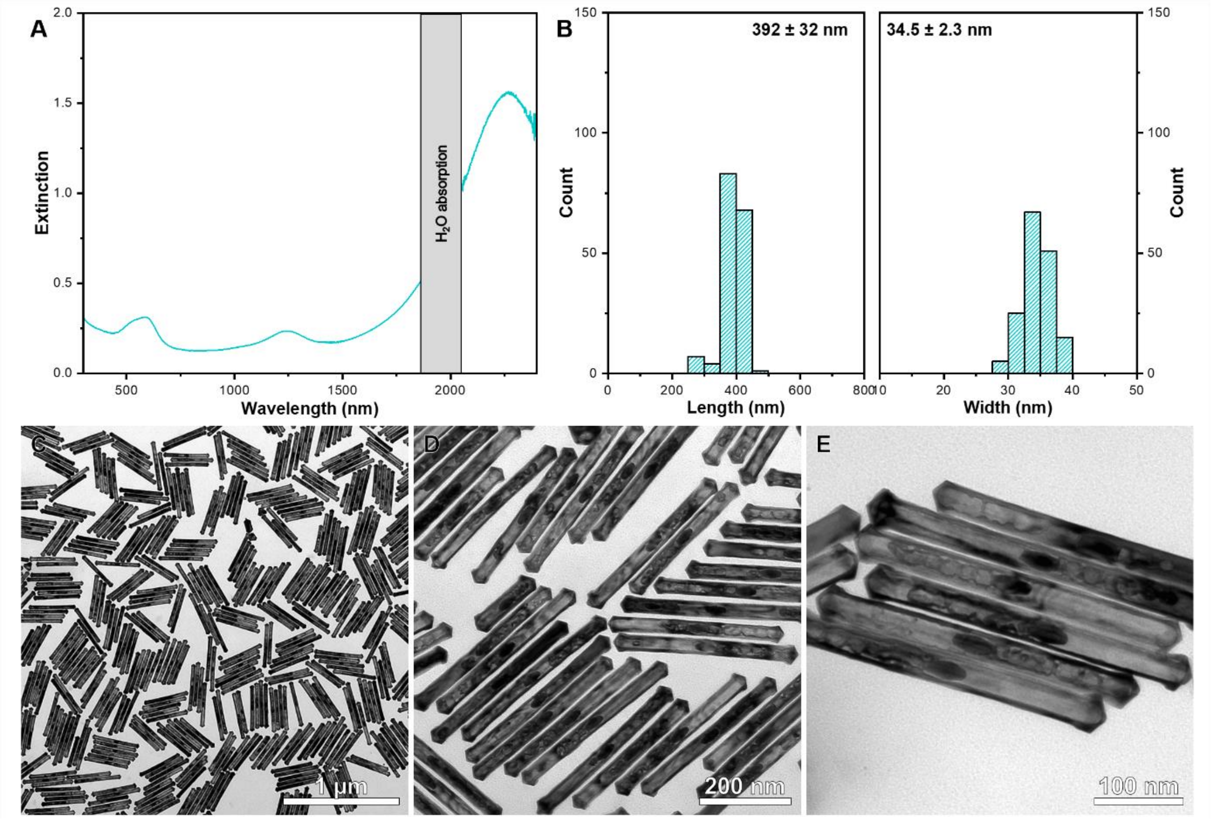

Figure S22. Characterization of the AuBP@AuAg NRTs employed for SEIRA. (**A**) UV-vis-NIR extinction spectrum of the gold nanoparticles in water. (**B**) Histogram obtained from the analysis of TEM images. (**C-E**) TEM images of AuBP@AuAg NRTs at different magnifications.

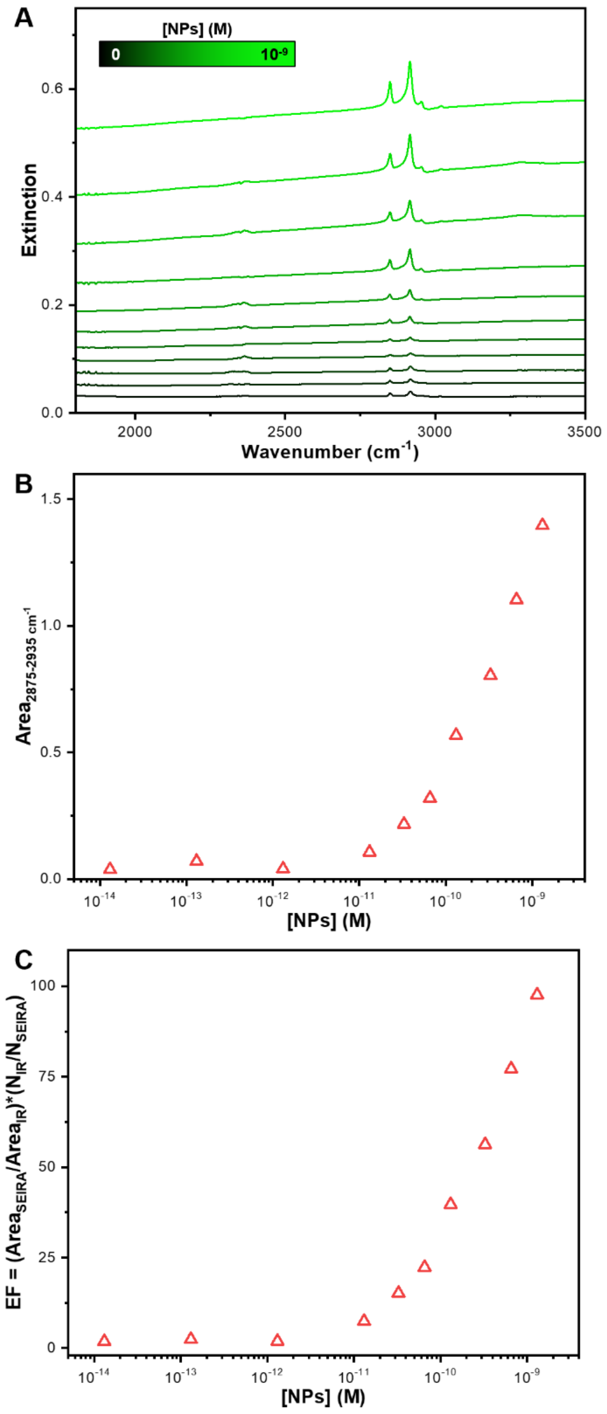

Figure S23. Evaluation of the SEIRA capabilities of AuBP@AuAg NRT. (**A**) Extinction SEIRA spectra of 10^{-4} M CTAB obtained in the presence of different nanoparticle concentrations of AuBP@AuAg NRTs, ranging from 10^{-14} M to 10^{-9} M. The IR spectrum without NTRs is included for comparison (**B**) Area of the characteristic SEIRA CTAB peak (2875-2935 cm^{-1}) as a function of molar nanoparticle concentration. (**C**) Enhanced factor (EF) as a function of molar nanoparticle concentration.
